# LoL-align: sensitive and fast probabilistic protein structure alignment

**DOI:** 10.1038/s41592-026-03249-w

**Published:** 2026-10-14

**Authors:** Lasse Reifenrath, Michel van Kempen, Gyuri Kim, Soo Hyun Kim, Mohammadreza Radnezhad, Cameron L. M. Gilchrist, Milot Mirdita, Martin Steinegger, Johannes Söding

**Affiliations:** 1 Quantitative and Computational Biology Group, Max Planck Institute for Multidisciplinary Sciences, Göttingen, Germany; 2 School of Biological Sciences, Seoul National University, Seoul, South Korea; 3 Interdisciplinary Program in Bioinformatics, Seoul National University, Seoul, South Korea; 4 Korea Basic Science Institute, Ochang-center, Cheongju, South Korea; 5 Department of Precision Medicine, Sungkyunkwan University School of Medicine, Suwon, South Korea; 6 Biomedical Institute for Convergence at Sungkyunkwan University, Sungkyunkwan University, Suwon, South Korea; 7 Artificial Intelligence Institute, Seoul National University, Seoul, South Korea; 8 Institute of Molecular Biology and Genetics, Seoul National University, Seoul, South Korea; 9 Campus Institute Data Science (CIDAS), Göttingen, Germany

**Keywords:** Software, Computational models

## Abstract

The ubiquitous availability of protein structures permits replacing sequence alignment with more accurate and sensitive structure alignment algorithms. LoL-align maximizes a local log-odds score for proteins to be homologous, given their intra-protein *C*_*α*_ − *C*_*α*_ distances. Here we show that LoL-align is markedly more sensitive in detecting remote homologs than TM-align and Dali on single- and multidomain benchmarks and achieves better alignment quality at 5–20 × their speed. LoL-align is fully integrated into Foldseek.

## Abstract (web summary)

LoL-align is a probabilistic, distance-based protein structure alignment algorithm that iteratively optimizes a local log-odds score, achieving higher sensitivity for remote homologs and better alignment quality than available methods.

## Main

Advances in protein structure prediction now yield models with near-experimental accuracy^1–4^. Today’s databases contain hundreds of millions of predicted structures^2,5^, which transforms large-scale protein analyses from sequence- to structure-based approaches and creates the need for accurate and efficient structural alignment tools.

Accurate structure aligners should detect remote homology with high sensitivity and specificity. They should produce accurate residue-level alignments, also for full-length multidomain proteins. The ability to detect local similarities and tolerance to conformational variability are essential. Speed is equally critical, as millions of structures need to be compared in large-scale analyses.

Existing methods meet these requirements only partially. TM-align^6^ and the fast, GPU-enabled GTalign superimpose proteins in three-dimensional (3D) space, offering accurate alignments for rigid, single-domain structures but limited tolerance for conformational flexibility. Dali^7,8^, which compares intramolecular distance patterns, remains among the most sensitive methods^9^, but its runtime precludes large-scale use. Foldseek^10^ compresses structures into a 20-state 3Di alphabet and applies MMseqs2-style searches^11^, achieving 10^4^–10^5^-fold speedups, but with reduced sensitivity and alignment quality. Several deep-learning-based approaches have been developed^12,13–15^, although they may struggle when applied to proteins outside their training distribution.

Here we present LoL-align, a distance-based algorithm that iteratively optimizes our new LoL-odds scoring function. The LoL-score quantifies the evidence for a pair of residues to be homologous given their *C*_*α*_-*C*_*α*_ distances and sequence distances within a 20Å neighborhood. LoL-align achieves higher sensitivity than Dali at 20 times higher speed. It is tolerant to conformational flexibility and produces more accurate alignments on average than TM-align and Dali. Integrated into Foldseek, LoL-align can refine top hits after prefiltering, enabling large-scale structural comparisons with high accuracy.

With LoL-align, we also present the first algorithm for single instruction multiple data (SIMD)-vectorized forward–backward probabilistic sequence alignment^16^, which achieved substantial performance gains through three optimizations: (1) SIMD-accelerated dynamic programming for parallel processing on modern CPU architectures; (2) a rescaling scheme to prevent numerical over- and underflow, which allows us to perform calculations in linear space without costly logarithmic transformations; and (3) a small memory footprint by overloading dynamic programming matrices.

**Fig. 1:**
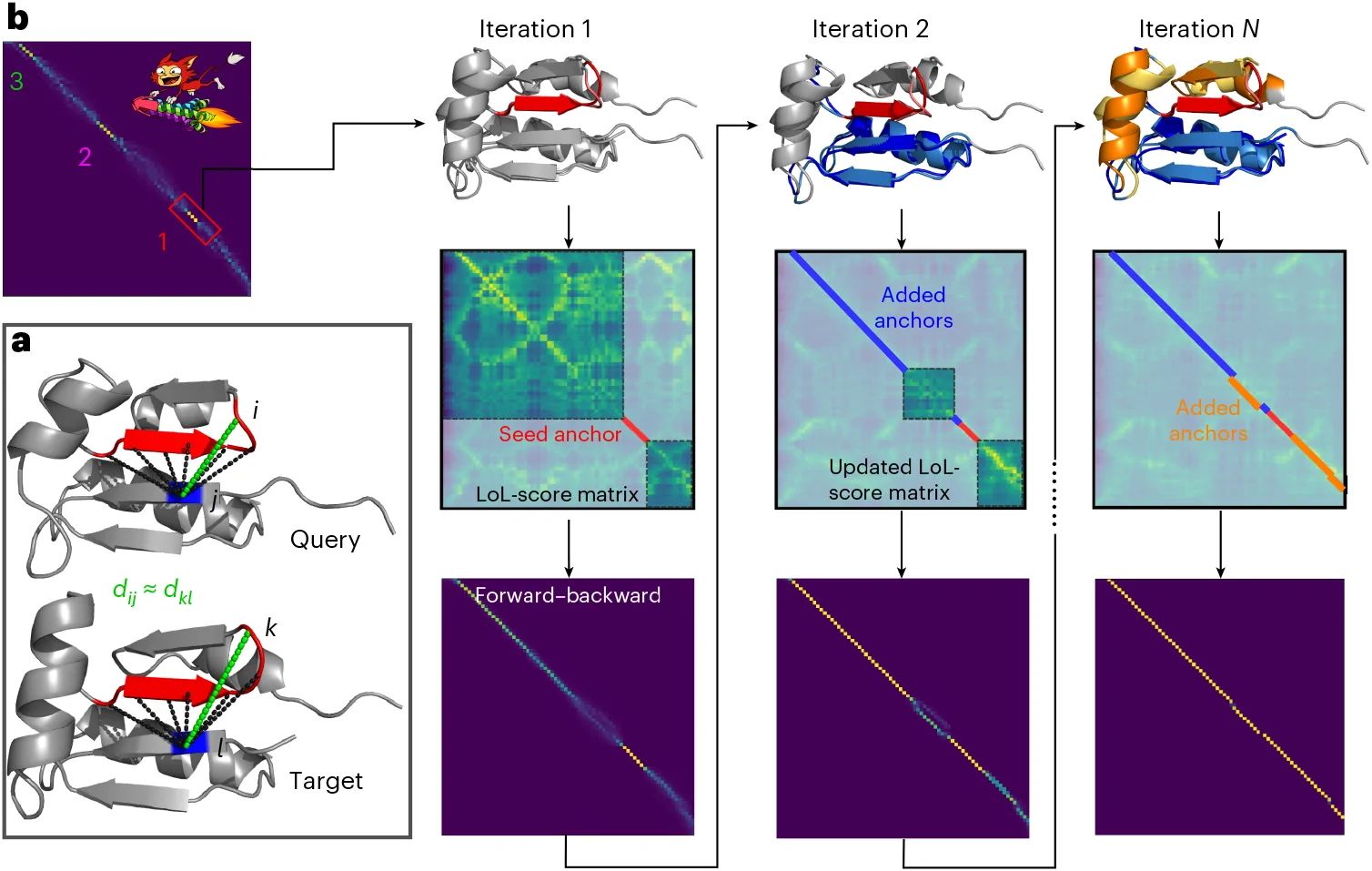
LoL-align algorithm. **a**, LoL-score. A candidate residue pair (*j*, *l*) (blue) is evaluated against a set *A* of aligned anchor pairs (*i*, *k*) (red). The LoL-score *S*_*jl*_ quantifies the evidence that *j* and *l* are homologous by comparing intramolecular *C*_*α*_-*C*_*α*_ distances in the query (*d*_*ij*_) and target (*d*_*kl*_) (green): ${S}_{jl}={\sum }_{(i,k)\in A}\log \frac{{p}_{ik,\;jl}}{1-{p}_{ik,\;jl}}$, where *p*_*ik*,*jl*_ is the probability that *i* is homologous to *k* and *j* to *l* given *d*_*ij*_, *d*_*kl*_, and *i* − *j* (Supplementary Fig. 1). **b**, Algorithm. Posterior alignment probabilities are computed from 3Di and amino acid substitution scores using our new vectorized forward–backward algorithm (top left). Three high-scoring seven-mers are chosen as seed anchors. Top row shows each seed anchor (here 1) is iteratively expanded (colored) until no further residues can be added. Middle row shows the LoL-scores are recomputed for all candidate residue pairs with the updated anchor set (colored lines). Bottom row shows posterior probabilities are computed, and pairs whose probability exceeds a threshold are added to the anchor set *A* (top row of next iteration).

LoL-align first runs the forward–backward algorithm using as match scores the sum of 3Di and amino acid substitution scores (Fig. 1). The top ten aligned seven-mers with the highest sum of posterior alignment probabilities are extended along the diagonal of the dynamic programming matrix, and the three top-scoring seven-mer seed anchors are retained for further refinement. For each seed anchor, the alignment is iteratively refined in three steps per iteration. First, a LoL-score matrix is computed using the current anchor pair residues. Second, posterior alignment probabilities are computed with the forward–backward algorithm. Third, residues exceeding a probability threshold are added to the set of anchor residues. In this way, the anchor residue set expands locally in 3D space until convergence is achieved (Fig. 1).

In single-domain mode, LoL-align divides the final scores by ${({L}_{Q}\times {L}_{T})}^{0.25}$, whereas in default multidomain mode, it uses no normalization. In this mode, if no new anchors are found, the *L*_*Q*_ × *L*_*T*_ 3Di and amino acid score matrices are added to the LoL-score matrix, and the forward–backward algorithm is rerun with this combined score. LoL-align can thereby align structures across structural domains even when they fall outside the 20 Å neighborhood of the anchor set. In single-domain mode, this step is omitted to reduce runtime.

**Fig. 2:**
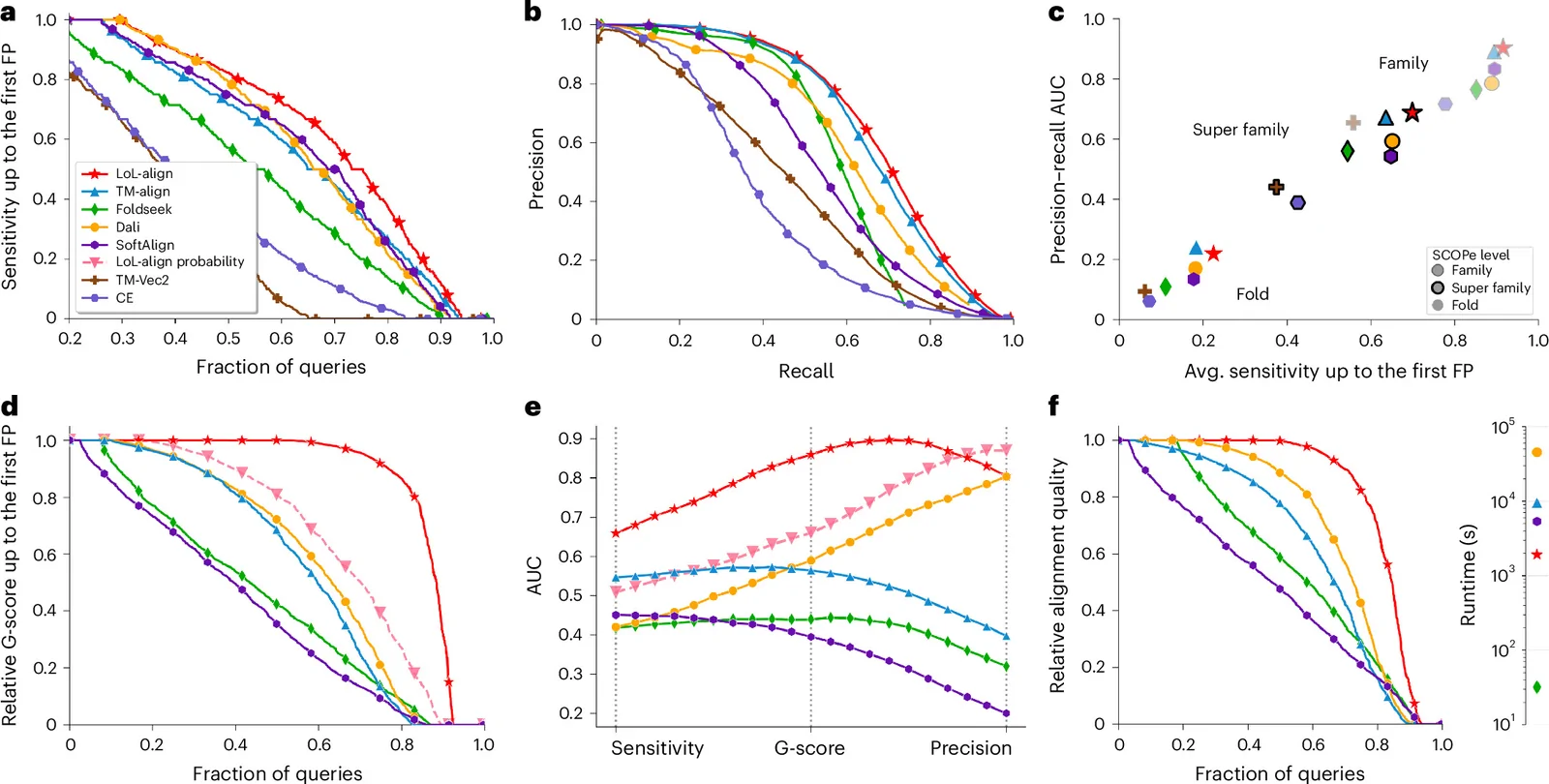
Search sensitivity and alignment quality. **a**–**c**, homology detection benchmark on the SCOPe40 single-domain structure database. Each structure is searched against all others. TPs are matches within the same superfamily, FPs are matches between different folds. **a**, Cumulative distributions of the sensitivity for ranking TPs before the first FP (see Supplementary Fig. 3 for family and fold). **b**, Precision versus recall for superfamily level detection (see Supplementary Fig. 2 for family and fold). **c**, Average sensitivity up to the first FP versus area under the precision–recall curve on family-, superfamily- and fold-level. **d**,**e**, Reference-free benchmark on full-length proteins from AFDB. The 2,000 queries sampled from AFDB were searched against up to 2,000 top hits found by Foldseek. **d**, Relative sensitivity up to the first FP, computed as the cumulative rescaled G-score divided by the maximum G-score per query across all methods. The G-score is the geometric mean of residue-wise precision (LDDT score normalized by the number of aligned residues) and sensitivity (LDDT score normalized by the number of query residues), rescaled to (0–1) for TPs; alignments with G-score <0.5 are classified as FPs. **e**, AUC evaluated across varying weights *α* that balance sensitivity and precision, calculated as precision^*α*^ × sensitivity^1−*α*^. **f**, Cumulative distribution of average alignment qualities, measured as the sum of residue-wise rescaled G-scores for all matches with G-score >0.5 divided by the maximum for the query across all tools. Inset shows total runtimes for all 2,000 searches. Source data

While the LoL-score reflects global similarity of two aligned proteins, it is less suited for determining whether a local alignment represents true homology. We therefore trained a small neural network on the Structural Classification of Proteins - extended (SCOPe) database to predict the probability of a homologous match from only local alignment features. On the single-domain benchmark, the probability performs similarly to the LoL-score (Supplementary Fig. 9), whereas on the full-length benchmark, it places more emphasis on residue-wise precision than sensitivity (Fig. 2d,e and Supplementary Fig. 4).

We calibrated specific LoL-score thresholds to correspond to defined precision levels for SCOPe superfamilies. For instance, a precision of 99% is achieved at a threshold of 0.65, a 95% precision is reached at 0.60, and a 75% precision at 0.50. A comprehensive list of these calibrated thresholds is provided in Supplementary Table 4.

## Results

We benchmarked the sensitivity of LoL-align and other popular structural aligners on the SCOPe database^17^. SCOPe classifies single structural domains into a hierarchy of families, superfamilies and folds. A nonredundant subset, SCOPe40 (11,211 proteins clustered at 40% sequence identity from SCOPe 2.01), was used for evaluation. The query set comprised 3,566 structures, restricted to SCOPe entries with at least one additional family-, superfamily- or fold-level member. For each query we perform a search against the whole database (11,211) and calculate the sensitivity up to the first false positive (FP), which is the fraction of true positives (TPs) found before the first FP in the list of ranked matches. TPs are defined to be from the same family, superfamily or fold and FPs are from different folds.

The cumulative distribution over all queries of sensitivities up to the first FP at the superfamily level in Fig. 2a shows that LoL-align has a 7.3% and 9.9% higher average sensitivity than Dali and TM-align, respectively (also Supplementary Fig. 3). In a precision–recall analysis, LoL-align attained an area under the curve (AUC) 14.5% higher than Dali and 2.3% higher than TM-align (Fig. 2b and Supplementary Fig. 2).

Searching large databases of full-length protein structures such as the AlphaFold Database^18^ (AFDB) is of critical importance nowadays. To assess the alignment quality and search sensitivity on full-length proteins, we sampled 2,000 query proteins from the 2.3 million cluster representatives in the clustered AFDB, requiring each protein to contain at least 100 residues with predicted local distance difference test (pLDDT) scores ≥ 0.7 (ref. ^1^). Each query was searched against the 2.3 million structures using Foldseek, retaining up to 2,000 top hits per query as the test set. Each tool was benchmarked by searching each query through these up to 2,000 hits.

For each alignment, we calculated its residue-wise precision, defined as LDDT score normalized by the number of aligned residues, and a residue-wise sensitivity, defined as LDDT score normalized by the number of query residues. We calculated the G-score as the geometric mean of precision and sensitivity, and classified alignments as TPs for LDDT G-score ≥0.5 and as true negatives otherwise (other cutoffs are shown in Supplementary Fig. 4). The G-scores were linearly rescaled to the range (0–1) (G-score 0.5 → 0, G-score 1.0 → 1.0). To compare the quality of matches per query on a linear scale, we defined the relative sensitivity for a query as the tool’s search sensitivity divided by the maximum sensitivity observed across all methods. LoL-align is considerably more sensitive than Dali and TM-align (Fig. 2d). The same trends hold when aligning structural models containing low-quality regions (Supplementary Fig. 5). Different alignment tools exhibit distinct preferences, with some favoring local, high-quality alignments (for example, Dali and LoL-align probability) over global structural similarities (for example, LoL-align and TM-align). This divergence directly impacts the trade-off between precision and sensitivity. To systematically evaluate this behavior, we tested varying weights (*α*) to balance sensitivity and precision, calculated as residue-wise precision^*α*^ × sensitivity^1−*α*^. For each *α* value, we computed the AUC of the resulting relative score (Fig. 2e). The reference-free nature forced us to assess search sensitivity and alignment quality in a single measure. To assess alignment quality without influence from sensitivity, we re-ranked all alignments for each query by their G-scores and plotted the cumulative distribution of relative rescaled G-scores (Fig. 2f and Supplementary Fig. 4), demonstrating that LoL-align produces alignments with higher G-scores than the other tools. Finally, on the AFDB benchmark, LoL-align was about five times faster than TM-align and 20 times faster than Dali.

### Discussion

LoL-align improves both sensitivity for remote homologs and alignment quality compared to state-of-the-art methods TM-align and Dali, for single domains and full-length proteins. The wide availability of structures for almost any full-length protein sequence calls for tools that combine sensitivity, speed and alignment accuracy with the ability to detect local similarities and align multidomain proteins. We demonstrated here that LoL-align addresses these needs by introducing a probabilistic structure similarity score and a new vectorized probabilistic sequence alignment algorithm to efficiently maximize this score. Integration into Foldseek enables systematic functional annotation and evolutionary analyses across hundreds of millions of predicted structures. LoL-align is freely available via the Foldseek web server (https://search.foldseek.com) or within the open-source Foldseek repo (https://github.com/steineggerlab/foldseek).

## Methods

### LoL-align algorithm overview

The LoL-align algorithm is illustrated in Fig. 1. First, the vectorized forward–backward algorithm computes for query and target proteins the posterior alignment probabilities for each pair of residues (*i*, *j*) based on the 3Di sequences. The top ten seven-mer seed anchor candidates with the highest posterior probabilities are re-ranked using gapless alignment centered on the seed using a combination of LoL-score, 3Di and amino acid scores (see ‘Seed anchors’ section). The top three candidates are used to initialize full alignments. For each seven-mer seed anchor, the algorithm proceeds iteratively:

1. Compute the matrix of LoL-scores using the current anchor residues.
2. Apply the forward–backward algorithm to obtain the posterior alignment probability matrix (*P*_*jl*_).
3. Add all residue pairs with ${P}_{jl}\ge {\mathop{\max }\limits_{ik}}({P}_{ik})-0.1$ to the set of anchor residue pairs.

The iteration terminates when no additional anchor pairs are detected. For the multidomain variant, if no new anchors are found, the 3Di and amino acid score matrices (each of dimension *L*_*Q*_ × *L*_*T*_, where *L*_*Q*_ and *L*_*T*_ denote the lengths of the query and target proteins, respectively) are added to the LoL-score matrix, and the iterative process is resumed from step 2. This allows LoL-align to extend alignments across multiple domains, even when the domains are spatially distant in 3D space.

### The LoL-score

The LoL-score quantifies the evidence that two residues are homologous by evaluating their intramolecular distance relationships relative to other aligned pairs. Let *d*_*ij*_ and *d*_*kl*_ denote the intramolecular *C*_*α*_ distances between residues *i* and *j* in the query and residue *k* and *l* in the target protein. The sequence distance in the query is denoted by *i* − *j*. Let *p*_*ik*,*jl*_ be the probability that residue *i* is aligned to residue *k* and *j* is aligned to *l*, given the distances *d*_*ij*_, *d*_*kl*_, and *i* − *j*. The residue-wise LoL-score *S*_*jl*_ with respect to a set of anchors *A* is defined as

$$S_{jl} = \sum\limits_{(i,k) \in A, \,d_{ij} \leq 20 {\mathring{{\rm{A}}}}} \ \log \frac{p_{ik,jl}}{1 - p_{ik,jl}}.$$

Here, $\log \frac{{p}_{ik,jl}}{1-{p}_{ik,jl}}$ is the log odds that a given residue pair originates from a homologous versus a nonhomologous alignment. We approximate this log odds function using a fully connected neural network function *g*(*d*_*ij*_, *d*_*kl*_, *i* − *j*) that takes (*d*_*ij*_, *d*_*kl*_, *i* − *j*) as input, includes a single hidden layer of dimension three with rectified linear unit activation, and outputs a single scalar value. Positive training distance pairs were sampled from Foldseek alignments within the same SCOPe superfamily, whereas negative distance pairs were sampled from alignments between proteins of different folds. As potential Foldseek misalignments constitute only a small fraction of the training data, the small network learns the dominant signal distinguishing homologous from nonhomologous residue pairs rather than fitting to these errors. The LoL-score for an entire alignment is the sum over the residue-wise LoL-scores for the aligned pairs: LoL-score(*A*) = ∑_(*j*, *l*)∈*A*_*S*_*jl*_.

The LoL-score matrix *S*_*jl*_ does not need to be computed for residue pairs that do not satisfy the strictly monotone alignment constraints (grayed out in Fig. 1). The LoL-scores can be updated incrementally when new anchor pairs *A*_new_ are added, reducing the computational cost of iterative refinement:

$${S}_{jl}\leftarrow {S}_{jl}+\sum _{(i,k)\in {A}_{{\rm{new}}},\,{d}_{ij}\le 20\,{\mathring{{\rm{A}}}}}\,\log \frac{{p}_{ik,jl}}{1-{p}_{ik,jl}}.$$

### Selection of new anchor points

Using the LoL-scores *S*_*ij*_ derived from the current set of anchor pairs *A*, the forward–backward algorithm is applied to compute the posterior alignment probability matrix *P*, where each element *P*_*jl*_ represents the probability that residues *j* and *l* are correctly aligned. New anchor pairs *A*_new_ are then selected as

$${A}_{\mathrm{new}}=\{(j,l)| {P}_{jl}\ge \max ({P}_{\max }-\delta p,\,{P}_{\min }),\,(j,l)\notin A\}$$

where ${P}_{\max }=\mathop{\max }\limits_{i,k}({P}_{ik})$ is the highest posterior probability in *P*, *δp* is a relative offset from this maximum (default 0.1), and ${P}_{\min }$ is the minimum probability threshold for inclusion.

### Vectorized forward–backward algorithm

The forward–backward algorithm is a widely used dynamic programming method for probabilistic protein sequence alignment. So far, the only implementations we are aware of are sluggish because they are unvectorized and they operate in log space to prevent numerical over- and underflows. The latter requires time-consuming log and exp operations to compute log-sum-exp operations. Here, we rewrite the update equations for the forward–backward algorithm in a way that allows for vectorization in Python or using the hardware-parallelized SIMD instructions available on modern CPUs. We also demonstrate how to perform the updates in linear space while avoiding under- and overflows.

#### The forward–backward algorithm

This computes from a score matrix *S*_*ij*_ the posterior probabilities *P*_*ij*_ that residue pair (*i*, *j*) is part of the alignment between a query and a target sequence with lengths *L*_*Q*_ and *L*_*T*_. Following Mueckstein et al.^16^, the probability *P*(*A*) of an alignment *A* is given by *P*(*A*) ∝ *e*^*βS*(*A*)^, with scaling constant *β* and alignment score *S*(*A*) = ∑_(*ij*)∈*A*_*S*_*ij*_ − affine gap costs. Normalization to 1 yields the probability distribution

$$P(A)=\frac{1}{Z}{e}^{\beta S(A)},$$

with normalization constant $Z={\sum }_{A\in {\mathcal{A}}}{e}^{\beta S(A)}$ (called partition function *Z*). The sum in *Z* runs over all possible alignments ${\mathcal{A}}$.

We define ${{\mathcal{A}}}_{ij}^{M}$ as the set of all alignments between residues 1: *i* from the query and 1: *j* from the target that end in (*i*, *j*) ∈ *A* (a ‘match’). Similarly, we define ${{\mathcal{A}}}_{ij}^{E}$ as the set of all alignments between residues 1: *i* from the query and 1: *j* from the target that end in a gap in the query protein, and analogously for ${{\mathcal{A}}}_{ij}^{F}$ ending with a gap in the target. We then define the partial partition functions ${Z}_{ij}^{X}={\sum }_{A\in {{\mathcal{A}}}_{ij}^{X}}P(A)$ with *X* = *M*, *E*, *F*. We denote the gap opening and extension penalties as *g*_o_ and *g*_ext_ (affine gap penalties). It can be shown^16^ that these three dynamic programming matrices can be iteratively computed in the forward pass,

1 $${Z}_{ij}^{M}=\left({Z}_{i-1,j-1}^{M}+{Z}_{i-1,j-1}^{E}+{Z}_{i-1,j-1}^{F}+1\right)\,{e}^{\beta {S}_{ij}},$$

2 $${Z}_{ij}^{E}={Z}_{i,j-1}^{M}{e}^{\beta {g}_{{\rm{o}}}}+{Z}_{i,j-1}^{E}{e}^{\beta {g}_{{\rm{ext}}}},$$

3 $${Z}_{ij}^{F}={Z}_{i-1,j}^{M}{e}^{\beta {g}_{{\rm{o}}}}+{Z}_{i-1,j}^{F}{e}^{\beta {g}_{{\rm{ext}}}}.$$

In the backward pass, we compute the matrices ${\hat{Z}}_{ij}^{M},{\hat{Z}}_{ij}^{E},{\hat{Z}}_{ij}^{F}$. Here, ${\hat{Z}}_{ij}^{M}$ is the sum of *P*(*A*) over all alignments ${\hat{{\mathcal{A}}}}_{ij}^{M}$ of residues *i*: *L*_*Q*_ in the query and *j*: *L*_*T*_ in the target starting with (*i*, *j*) ∈ *A*, and analogously for ${\hat{Z}}_{ij}^{E}$ and ${\hat{Z}}_{ij}^{F}$ starting with a gap in the query or template, respectively. The update equations for the backward pass are analogous to the ones for the forward pass^16^.

Using the matrices from the forward and backward pass, the posterior probability *P*_*ij*_ can be calculated by

$${P}_{ij}=\sum _{A:(i,j)\in A}P(A)=\frac{1}{Z}\sum _{A:(i,j)\in A}{e}^{\beta S(A)}=\frac{{e}^{-\beta {S}_{ij}}}{Z}\sum _{A\in {{\mathcal{A}}}_{ij}^{M}}{e}^{\beta S(A)}\sum _{A\in {\hat{{\mathcal{A}}}}_{ij}^{M}}{e}^{\beta S(A)}=\frac{{e}^{-\beta {S}_{ij}}}{Z}{Z}_{ij}^{M}{\hat{Z}}_{ij}^{M}.$$

#### Vectorized forward–backward

We introduce a new normalization scheme and reformulate key computations to ensure numerical stability in linear space while enabling efficient SIMD vectorization. We divide the target protein into *b* blocks of length *L*. Because the alignment score is additive, the calculation for ${Z}_{i,j}^{M}$, ${Z}_{i,j}^{E}$, and ${Z}_{i,j}^{F}$ depend only on values from the preceding block (*b* − 1). Each block *b* can therefore be fully processed once the previous block is complete. Within each block, every row *i* is rescaled by $\max \{\mathop{Z}\nolimits_{i,j}^{M}:(b-1)L < j\le bL\}$ to prevent numerical over- or underflow along the query dimension. These scaling factors are stored in log space, while all other operations are performed in linear space. To maintain continuity between blocks, we store the final values of ${Z}_{i,bL}^{M}$, ${Z}_{i,bL}^{E}$, and ${Z}_{i,bL}^{F}$ in a compact buffer and use them to initialize the corresponding entries of the next block. These values are updated and then reused as the starting conditions for the next block, ensuring continuity without re-initialization while preventing over- and underflows. The rescaling parameters are incorporated into the final ${Z}_{ij}^{M}$ when calculating the posterior probability matrix *P*_*ij*_.

By setting the block length *L* to match the SIMD register width (for example, *L* = 8 on AVX2 architectures), one operation for a whole line of a block can be processed simultaneously using SIMD vectorization. The recursions for ${Z}_{ij}^{M}$ and ${Z}_{ij}^{F}$ are trivial to vectorize, as they depend only on the preceding row. In contrast, ${Z}_{ij}^{E}$ depends on its immediate left neighbor, which prevents direct vectorization. By applying equation (2) iteratively, we obtain

$$\begin{array}{l}{Z}_{i,k}^{E}={Z}_{i,k-1}^{M}{e}^{\beta {g}_{{\rm{o}}}}+{Z}_{i,k-1}^{E}{e}^{\beta {g}_{{\rm{ext}}}}\\\qquad={Z}_{i,k-1}^{M}{e}^{\beta {g}_{{\rm{o}}}}+\left({Z}_{i,k-2}^{M}{e}^{\beta {g}_{{\rm{o}}}}+{Z}_{i,k-2}^{E}{e}^{\beta {g}_{{\rm{ext}}}}\right){e}^{\beta {g}_{{\rm{ext}}}}\\\qquad=\ldots \\\qquad=\mathop{\sum }\limits_{j=0}^{k-1}\left({Z}_{i,j}^{M}\,{e}^{\beta (k-1-j){g}_{{\rm{ext}}}+\beta {g}_{{\rm{o}}}}\right)+{Z}_{i,0}^{E}{e}^{\beta k{g}_{{\rm{ext}}}}\\\qquad={e}^{\beta k{g}_{{\rm{ext}}}}\left(\mathop{\sum }\limits_{j=0}^{k-1}\left({Z}_{i,j}^{M}\,{e}^{\beta (-1-j){g}_{{\rm{ext}}}+\beta {g}_{{\rm{o}}}}\right)+{Z}_{i,0}^{E}\right).\end{array}$$

With the precomputed, constant vectors ${V}_{[0:L-1]}={e}^{-\beta {g}_{{\rm{ext}}}[1:L]+\beta {g}_{{\rm{o}}}}$ and ${W}_{[0:L-1]}={e}^{\beta {g}_{{\rm{ext}}}[1:L]}$, the ${Z}_{ij}^{E}$ in the first block (*b* = 1) of indices [1:L] can be updated in vectorized fashion,

$${Z}_{i,[1:L]}^{E}={W}_{[0:L-1]}* \left(\,\mathrm{CumSum}\,\left({Z}_{i,[0:L-1]}^{M}* {V}_{[0:L-1]}\right)+{Z}_{i,0}^{E}\right),$$

where * denotes element-wise multiplication of vectors. The update equation for later blocks *b* is the same except for an offset (*b* − 1)*L* on all *j* indices.

#### Fast C++ SIMD implementation

To minimize memory usage, a single dynamic programming buffer matrix is reused across all stages of the algorithm. During the forward pass, log-values are calculated from the alignment score matrix and written into the matrix. In the backward pass, these entries are read into SIMD vectors, added with backward contributions in log space, and written back to the same locations, eliminating the need for a separate backward matrix. Finally, in the posterior calculation step, the matrix is directly aliased as probability matrix *P* and overwritten with posterior probabilities after normalization and lin-space transformation, again in SIMD vectors. All block-level scratch buffers are similarly recycled across passes, so every update happens in-place with vectorized loads and stores, reducing memory traffic and avoiding redundant allocations.

We implemented SIMD-optimized logf and expf approximations, based on Agner Fog’s Vector Class Library^19^, for accelerated evaluation of transcendental functions on 32-bit floats. Both methods apply range reduction followed by minimax polynomial approximation, and compute 4–8 values per instruction using streaming SIMD extensions (SSE) or advanced vector extensions (AVX) registers. The logf implementation extracts the IEEE-754 mantissa and exponent to evaluate $\ln (x)\approx e\cdot \ln 2+\ln (1+m)$ using an eighth-order polynomial on a normalized mantissa. The expf implementation performs argument reduction via ${\log }_{2}(e)$ and reconstructs the result using a fifth-order polynomial and fast exponent bit synthesis (2^*r*^). The implementations are branch-free aside from masked special-case handling, enabling substantial throughput gains over scalar libm functions while maintaining accuracy suitable for numerically sensitive bioinformatics workloads.

### Seed anchors

The calculation of the LoL-score matrix relies on identifying correctly aligned residue pairs, termed anchors. As seed anchors, we selected high-confidence seven-mers. Anchors were identified using a composite score that combines Foldseek’s 3Di and BLOSUM62 substitution scores, weighted 2.1 and 1.4, respectively, following the Foldseek methodology^10^. To verify that this weighting does not overfit to a particular dataset, we evaluated alternative ratios on SCOPe (Supplementary Table 2). Furthermore, as the original 3Di substitution matrix was fitted on SCOPe and assigns high scores to matches in unstructured regions, we instead use a 3Di matrix re-fitted on TM-align alignments from the AlphaFold database, which yields lower scores for such spurious matches (Supplementary Table 5). Posterior alignment probabilities were computed with the vectorized forward–backward algorithm using hyperparameters gap opening = 6, gap extension = 3 and temperature = 2. Anchors were defined as the residue pair with the highest posterior probability, together with its three upstream and downstream neighbors. This way, ten nonoverlapping seven-mers were chosen as candidate seed anchors and ranked according to the score of the best ungapped alignment passing through their central position. Ungapped alignments were computed using both the composite score (3Di and amino acid) and the LoL-score matrix, with the corresponding seven-mers serving as anchors. The three top-ranked sets were subsequently refined in the iterative alignment process (Fig. 1). Because the final alignment depends on the selected anchors, we expose two user-configurable parameters: the number of candidate seeds for which an ungapped structure alignment is computed (--candidate-seeds) and the number subsequently refined top-ranked seeds (--refine-seeds). The effect of --refine-seeds is reported in Supplementary Table 3 and Supplementary Figs. 4 and 5.

### Score calculation

For ranking alignments, we combine the LoL-score, the 3Di score and the amino acid BLOSUM62 score. To correct for scale differences between residues, we introduce a rescaling factor derived from a self-hit score for each aligned query residue *i* :

$${S}_{i}^{{\prime} }=\sum _{j\in A,\,{d}_{ij} < 20{\mathring{{\rm{A}}}}\,}g({d}_{ij},{d}_{ij},i-j).$$

This value represents the maximal LoL-score contribution of residue *i* within an alignment. The rescaling factor is then defined as the mean ratio between observed and optimal contributions:

$$\,\mathrm{LoL-rescale}\,=\frac{1}{| A| }\sum _{(i,k)\in A}\frac{{S}_{i,k}}{{S}_{i}^{{\prime} }}.$$

The raw alignment score is multiplied by this factor (where AA is amino acid):

$$\begin{array}{l}{\mathrm{Alignment}}\mbox{-}{\mathrm{score}}\,(A)=\left(\,{\mathrm{LoL}}\mbox{-}{\rm{score}}(A)+6.3\times {\mathrm{3Di}}(A)\right.\\\left.\qquad\qquad\qquad\qquad\quad+4.2\times {\mathrm{AA}}(A)\right)\times {\mathrm{LoL}}\mbox{-}{\mathrm{rescale}}.\end{array}$$

This score is not comparable across queries, as it depends on protein compactness and length. To address this, we normalize by the query–query (QQ) alignment score. In the single-domain version, we additionally normalizes by length by dividing by ${({L}_{Q}\times {L}_{T})}^{0.25}$:

$$\begin{array}{l}\mathrm{Alignment-Score}\,(A)\\=\sqrt{\left(\,\mathrm{LoL-score}(A)+6.3\times \mathrm{3Di}(A)+4.2\times \mathrm{AA}(A)\right)\times \frac{\mathrm{LoL-rescale}}{\mathrm{QQ-Score}\,\times {({L}_{Q}{L}_{T})}^{0.25}}}.\end{array}$$

Length normalization improves ranking of single-domain proteins, but can obscure homologous matches in multidomain proteins. Therefore, in the multidomain version, the ${({L}_{Q}{L}_{T})}^{0.25}$ factor is omitted.

### Homology probability prediction

To refine search results beyond raw geometric similarity, a small feed-forward neural network estimates the probability that an aligned protein pair is homologous. The network leverages length-independent local alignment features, including structural scores, contact-graph topology and local neighborhood preservation. Using only local features ensures that the predicted probabilities work in exactly the same way on full-length, multidomain proteins as on the single-domain SCOPe training set.

#### Training dataset and label formulation

Training pairs were generated via an all-against-all alignment of the SCOPe40 domain dataset (*n* = 11,211)^17^. For each ordered pair (*Q*, *T*), shared families or superfamilies were labeled as positives, distinct folds as negatives, and same-fold/different-superfamily pairs (remote homology) were excluded. To address the severe class imbalance, we randomly downsampled the negative pairs to a 1% subset per query. The remaining imbalance was then compensated for by weighting the loss function during training. Models were evaluated using fivefold cross-validation with a fold-disjoint split, ensuring that no SCOPe fold was shared between training and validation partitions. Fivefold cross-validation results on the SOCPe database are presented in Supplementary Fig. 9.

#### Feature extraction

Structural alignments were mapped to a 40-dimensional feature vector spanning three modalities:

1. Alignment scalars (8): raw and log-transformed LoL-score and LoL-rescale scores, alignment LDDT^20^, log-transformed alignment length and gap-open ($\frac{{g}_{{\rm{open}}}}{| A| }$) and gap-residue ($\frac{{g}_{{\rm{res}}}}{| A| }$) fractions, where ∣*A*∣ is the number of aligned columns.
2. Aligned-neighbor fraction (14): derived from a C*α* contact graph (Euclidean distance *d*_*ij*_ < 20 Å). For each aligned residue *r*, the aligned-neighbor fraction (ANF) is defined as:
  4 $${\rm{ANF}}(r)=\frac{a(r)}{d(r)+5},$$
  where *d*(*r*) is the total residue degree, *a*(*r*) is the number of its neighbors present in the alignment, and we use five pseudocounts to push ANF down for residues with few neighbors. Residue ANF values are summarized into a normalized seven-bin histogram (boundaries 0, 0.25, 0.5, 0.6, 0.7, 0.8, 0.9 and 1.0) independently for query and target domains.
3. Sequence-separation topology (18): contact-graph sequence separations ∣*i* − *j*∣ are partitioned into six bins (boundaries 5, 13, 31, 51 and 101) to compute three six-dimensional descriptors over aligned residues: tamax, the per-bin maximum absolute difference between query and target separation profiles; qsep, the mean sequence-separation profile of aligned query residues; and qfrac, the fraction of query contacts with both end points preserved in the alignment.

#### Network architecture and optimization

The classifier is a multilayer perceptron with two hidden layers (32 and 16 units), each utilizing rectified linear unit activation and dropout (*P* = 0.1). Input features are *z*-score standardized using training set statistics. The output layer maps the linear logit *z* to a probability for homology via the logistic sigmoid function: $P(\mathrm{homology})=\sigma (z)={(1+{e}^{-z})}^{-1}$.

Training proceeded for 15 epochs minimizing binary cross-entropy loss with a positive-class weight (≈2.7). Optimization used the AdamW optimizer^21^ (initial learning rate 10^−3^, weight decay 10^−4^, batch size 4,096 and gradient clipping norm 1.0) under a cosine-annealing schedule. The final parameter checkpoint was selected by maximizing the validation area under the receiver operating characteristic curve.

### Residue-wise sensitivity, precision and LDDT G-score

The LDDT^22^ is a popular measure to assess the quality of protein structure models. It addresses key limitations of older scoring methods such as root mean square deviation, global distance test score and TM-score that relied on global structure superposition, making them not well suited for assessing models of proteins that can undergo conformational changes or of multidomain proteins with poorly defined relative orientations of their domains. LDDT’s recently exploded popularity stems from its use by AlphaFold2 and other deep-learning protein structure predictors, which predict together with the protein structure the LDDT score for each residue (predicted LDDT; pLDDT) as a local measure of model accuracy. The LDDT score evaluates each aligned pair of residues $(i,k)\in {\mathcal{A}}$ by a residue-wise score between 0 and 1 that describes how well the distances of residues in one structure to its nearest spatial neighbor residue *j* agree with the distances between the aligned residues in the aligned (model) structure. Each pair of homologous distances (*d*_*ij*_, *d*_*kl*_) obtains a residue-wise score between 0 and 1, 1 for very good agreement (*d* = *d*_*ij*_ − *d*_*kl*_ < 0.5Å), 0.75, 0.5 and 0.25 for partial agreement, and 0 for a deviation of more than 4Å. The residue-wise score for residue pair (*i*, *k*) is the mean of these *N*_*i*_ distance scores. The LDDT score is the sum of residue-wise scores for all aligned pairs divided by the number of query residues *L*_*q*_:

$$\mathrm{LDDT}\,(A)=\frac{1}{{L}_{q}}\sum _{(i,k)\in {\mathcal{A}}}\frac{1}{{N}_{i}}\sum _{(j,l)\in A\,{d}_{ij}\le 15,{\mathring{{\rm{A}}}}}{g}_{{\rm{LDDT}}}(| {d}_{ij}-{d}_{kl}| ),$$

where ${\mathcal{A}}$ is the set of aligned residues, *N*_*i*_ is the number of aligned neighbors within 15 Å, and, *g*_LDDT_ is a step function that scores the difference *d* between two intramolecular distances,

$${g}_{{\rm{LDDT}}}(d)=\frac{1}{4}({\mathbb{1}}(d\le 0.5)+{\mathbb{1}}(d\le 1.0)+{\mathbb{1}}(d\le 2.0)+{\mathbb{1}}(d\le 4.0)).$$

The LDDT score thus answers the question what fraction of query residues was aligned correctly to the target structure, while considering a continuous measure for correctness in the form of the residue-wise LDDT score. When using it as a score for the quality of a structural alignment, it therefore measures residue-wise sensitivity (also called ‘recall’); the fraction of query residues that was actually correctly aligned. Taken alone, it favors overprediction of aligned residue pairs because it does not penalize overpredictions.

To address this, we could normalize the LDDT score by the number of aligned residues $| {\mathcal{A}}|$ instead of the number of query residues. This score measures the fraction of aligned query residues that was actually correctly aligned (the residue-wise precision). Taken alone, it favors underprediction, omitting all but the most reliable parts of the alignment.

Neither sensitivity nor precision alone are a good measure of performance as we need both to be high for an alignment to be useful. A common balanced measure is the F1 score, equal to the harmonic mean of sensitivity and precision; however, as our precision is always greater than or equal to sensitivity, the F1 score can disproportionately emphasize sensitivity. We therefore chose the G-score, the geometric mean of precision and sensitivity.

When working with protein structures predicted by AlphaFold, mispredicted regions must be handled carefully. Residues in such regions typically have low pLDDT scores. Therefore, we used only residues with pLDDT ≥ 0.5 to calculate the LDDT score and adjusted both the query and alignment lengths accordingly when normalizing the score.

### Full-length benchmark

To assess performance on full-length proteins, we sampled 2,000 cluster representatives from the clustered AlphaFold database^18^, selecting only proteins with at least 100 residues scoring ≥0.7 pLDDT. To evaluate performance on low-confidence regions, we additionally sampled 1,000 queries containing at least 100 residues with pLDDT≤0.5. Results for the high- and low-confidence sets are reported in Supplementary Figs. 4 and 5, respectively. Each query was searched against all representatives using Foldseek (parameters: -s 9.5 -e 10 --max-seqs 2,000). The resulting hits were aligned with Dali, TM-align, LoL-align and Foldseek, and ranked by their respective scores. For TM-align, we used the TM-score normalized by query length, which performed best on this benchmark. LoL-align was used in the multidomain version (--lolalign-multidomain 1).

For each alignment, we calculated the residue-wise precision and sensitivity. Alignments with an LDDT G-score ≥0.5 were classified as TPs (Supplementary Fig. 4 lists the alternative thresholds). To give very good alignments more weight than marginally correct ones, G-scores were linearly rescaled from (0.5−1) to the range (0−1) via Score = 2 (G-score −0.5). We measured search sensitivity as the cumulative rescaled G-score up to the first FP, including self-hits to avoid zero scores in log space. We then defined relative sensitivity as this search sensitivity divided by the maximum sensitivity across all methods for that query; here, we exclude self-hits (Fig. 2d). To evaluate the trade-off between precision and sensitivity, we calculated the AUC of the relative sensitivity across varying parameter weights (*α*) using precision^*α*^ × sensitivity^1−*α*^, where *α* = 0.5 corresponds to the standard G-score. Finally, to assess alignment quality independently of method-specific ranking, we ranked all alignments by their G-scores (excluding self-hits) and plotted the cumulative distribution of relative rescaled G-scores (Fig. 2f).

We note that LoL-align and Dali optimize scores more closely related to the LDDT metric, as all are based on comparing intramolecular distances, in contrast to TM-align. We selected the LDDT-based G-score because metrics requiring structural superposition, such as TM-score or root mean square deviation, do not adequately capture alignment quality for full-length, multidomain proteins and larger proteins with conformational flexibility, which were the focus of this reference-free benchmark.

All alignment tools were executed on a dual-socket AMD EPYC 7742 system with 128 physical cores. Tools with native multi-threading support (LoL-align and Foldseek) were run with 128 threads. Tools without multi-threading support were parallelized by splitting the query set into 128 equally sized batches and processing them in parallel.

### SCOPe benchmark

For the SCOPe benchmark, alignment results for Dali, TM-align and Foldseek were reused from the Foldseek study^10^, where the details of the alignment procedures are described. LoL-align was applied in the single-domain setting (--lolalign-multidomain 0).

#### Evaluation SCOPe benchmark

After sorting the alignment results for each query, sensitivity was calculated as the fraction of TPs in the ranked list up to the first FP, excluding self-hits. Mean sensitivity was compared across all queries at the family, superfamily and fold levels. Only SCOPe entries with at least one additional family-, superfamily- or fold-level member were included. Sensitivity was also measured up to the first FP (ROC1), rather than to the *k*-th FP (for example, the fifth FP), as ROC1 better reflects the requirements of low false discovery rates in automatic searches. We also analyzed the performance on different sequence identity levels (Supplementary Figs. 6–8).

Precision–recall curves were generated for each tool. Alignment results were sorted by structural similarity scores, and self-matches were excluded. Curves were computed separately at the family, superfamily and fold levels, with precision defined as TP/(TP + FP) and recall as TP/(TP + FN) (FN, false negatives). All counts (TP, FP and FN) were weighted by the reciprocal of their respective family, superfamily or fold size, ensuring that families, superfamilies and folds contributed linearly with their size rather than quadratically.

### LoL-align usage

LoL-align can be used within Foldseek’s easy-search by setting –alignment-type 3, which runs Foldseek’s search (3Di prefilter and 3Di + amino acid alignment) and realigns the top hits using LoL-align. To use LoL-align without Foldseek’s prefilter, a fake prefilter can be created as described in the MMseqs2 user guide, and the alignment can then be executed with Foldseek lolalign queryDB targetDB prefilter results.

On the Foldseek server, LoL-align can be used to realign search hits by selecting the LoL-align option under ‘Databases and search’ settings.

The integration of LoL-align into Foldseek facilitates its integration with other tools within the Foldseek ecosystem, such as Foldmason^23^, for which replacing TM-align by LoL-align resulted in slight improvements (Supplementary Table 1).

### Reporting summary

Further information on research design is available in the Nature Portfolio Reporting Summary linked to this article.

## Online content

Any methods, additional references, Nature Portfolio reporting summaries, source data, extended data, supplementary information, acknowledgements, peer review information; details of author contributions and competing interests; and statements of data and code availability are available at https://doi.org/10.1038/s41592-026-03249-w.

## Supplementary information

Supplementary Information Supplementary Figs. 1–9 and Tables 1–5.

Reporting Summary

Peer Review file

## Source data

Source Data Fig. 2 Source data underlying Fig. 2a–c (SCOPe40 benchmark: sensitivity, precision–recall and AUC) and Fig. 2d–f (AFDB benchmark: relative sensitivity, alpha-sweep AUC and alignment quality).

## Data Availability

Data utilized to perform benchmarks for this study are freely available at https://wwwuser.gwdguser.de/~compbiol/lolalign/. Source data are provided with this paper.

## Code availability

All code developed in this study is available under GPLv3 license and documented at https://foldseek.com/. The SIMD Forward–Backward algorithm is implemented in MMseqs2 under MIT license at mmseqs.com. Analysis scripts are available at https://github.com/soedinglab/LoLalign-analysis.
