# Supplementary material for: LoL-align: sensitive and fast probabilistic protein structure alignment
Source: Nat Methods. 2026 Oct 14;23(11):2147–50. doi: 10.1038/s41592-026-03249-w (PMC13736684; doi:10.1038/s41592-026-03249-w)
Supplement: Supplementary file 1 [file 41592_2026_3249_MOESM1_ESM.pdf]

nature methods

Supplementary information https://doi.org/10.1038/s41592-026-03249-w

LoL-align: sensitive and fast probabilistic protein structure alignment

In the format provided by the authors and unedited

Supplementary information

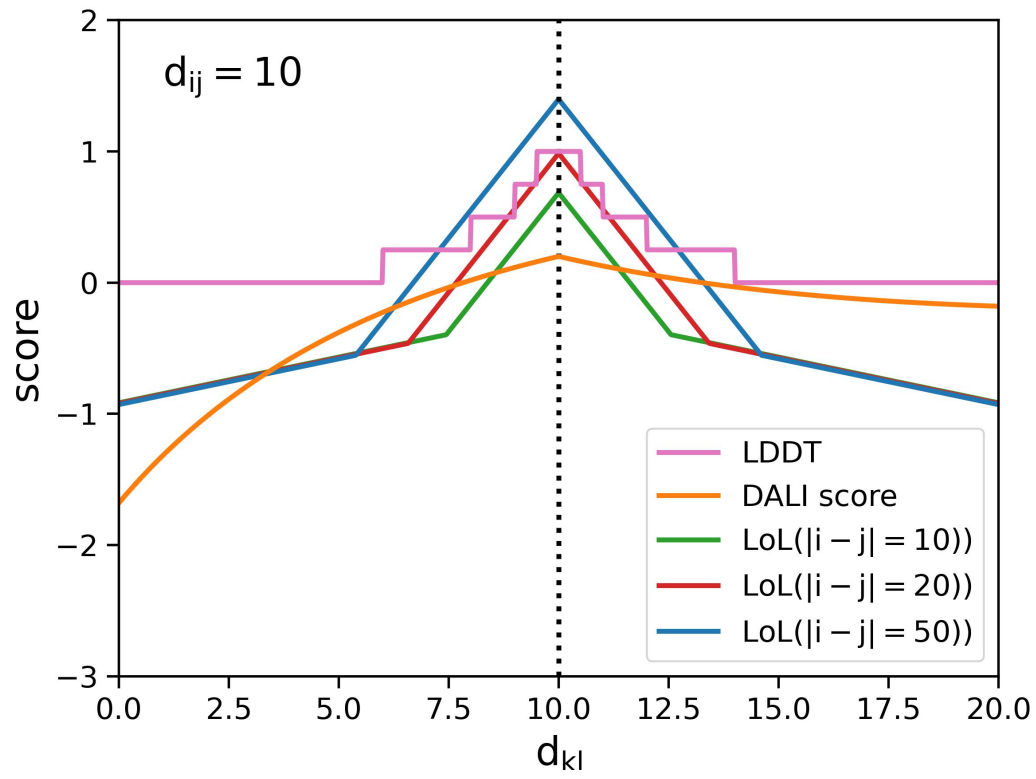

Figure 1: **Visualization of the LoL-Score.** Visualization of the LoL-score with different sequence distances as well as the Dali- and LDDT-score.

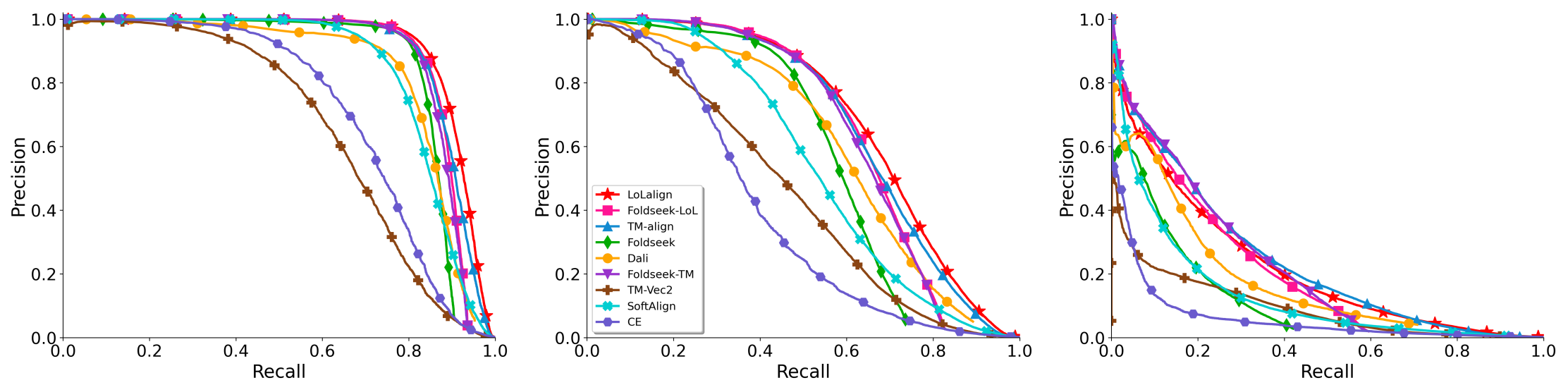

Figure 2: **Precision-Recall curves on SCOPe benchmark** We measured the sensitivity on the SCOPe40 dataset by plotting precision-recall curves on family, superfamily, and fold level. Where Foldseek-TM and Foldseek-LoL uses the Foldseek search with `-s 9.5 -e 10 --max-seqs 4000` as a prefilter.

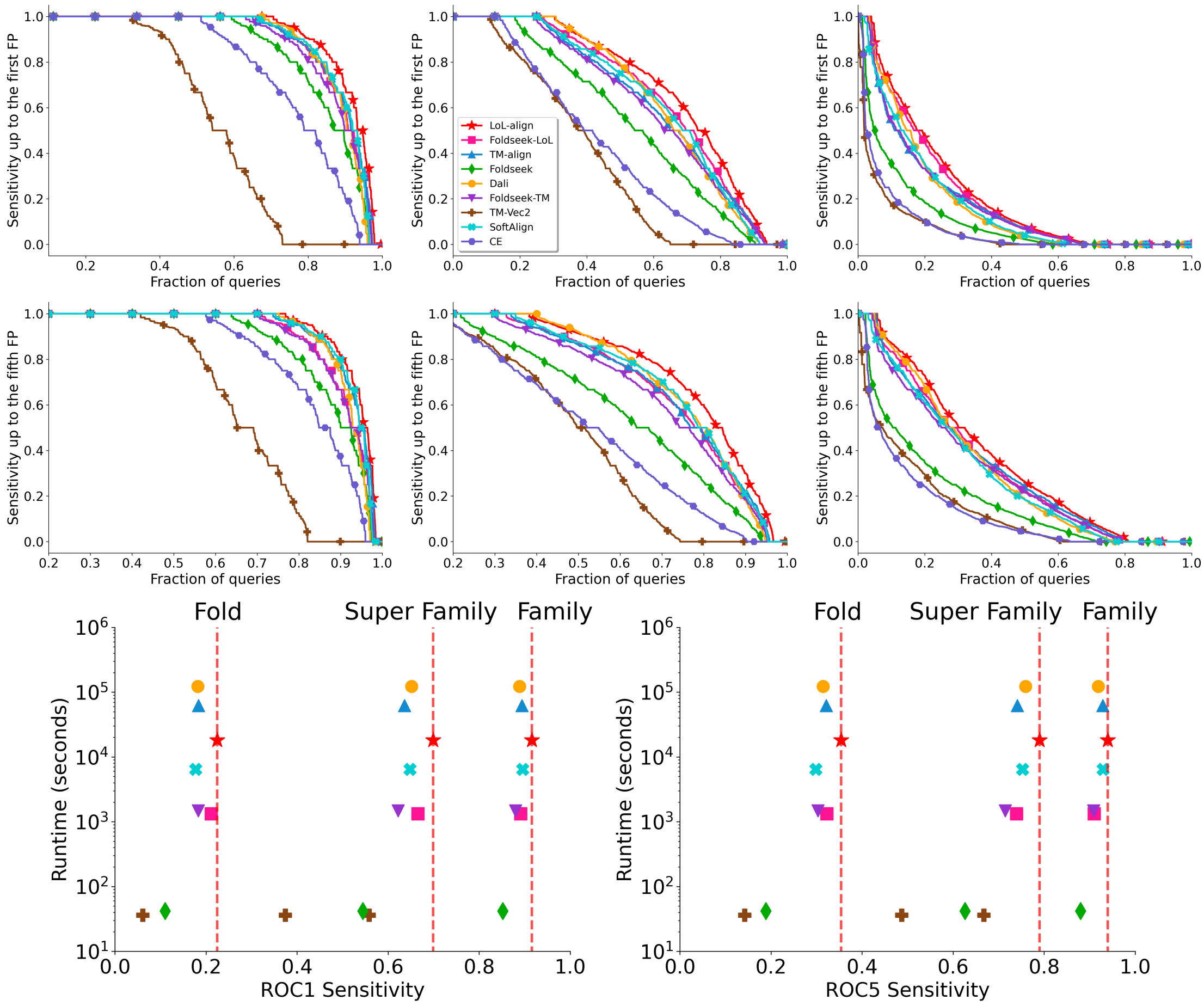

Figure 3: **cumulative distributions of sensitivity for the SCOPe benchmark** We measured the sensitivity on the SCOPe40 dataset by plotting the cumulative distributions of sensitivity up to the first and fifth FP on family, superfamily, and fold level. Here, Foldseek-TM and Foldseek-LoL use the Foldseek search with `-s 9.5 -e 10 --max-seqs 4000` as a prefilter. We also show the average ROC1 and ROC5 sensitivity plotted against the runtime. For TM-align we used the `-fast` parameter.

Method	homstrad			afdb			overall
	prec	sens	f1	lddt	osc	f1	f1
+LoLalign	78.2145	78.4109	78.3126	60.3907	65.1144	62.6637	69.6196
Original	77.9218	78.0327	77.9772	60.4139	65.145	62.6903	69.5031

Table 1: Benchmark performance of Foldmason when LoL-align is incorporated into the initial alignment stage.

	Family	Super Family	Fold
1.4:1.4	0.885364	0.660518	0.214206
1.4:2.1	0.891	0.66584	0.2112
2.8:1.4	0.884126	0.659299	0.213696

Table 2: Comparison of 3Di-to-amino-acid scoring ratios used for seed anchor identification, with results computed using the Foldseek prefilter. Shown is the mean ROC1 sensitivit on Family, Super Family and Fold Level.

	Family	Super Family	Fold
1	0.903242	0.665046	0.204778
2	0.90952	0.684695	0.219422
3	0.91302	0.694416	0.226567
5	0.915046	0.70199	0.232288
10	0.916898	0.707622	0.239391

Table 3: Comparison of `--refine-seeds` parameter settings in an all-vs-all search using the SCOPe dataset. Shown is the mean ROC1 sensitivit on Family, Super Family and Fold Level.

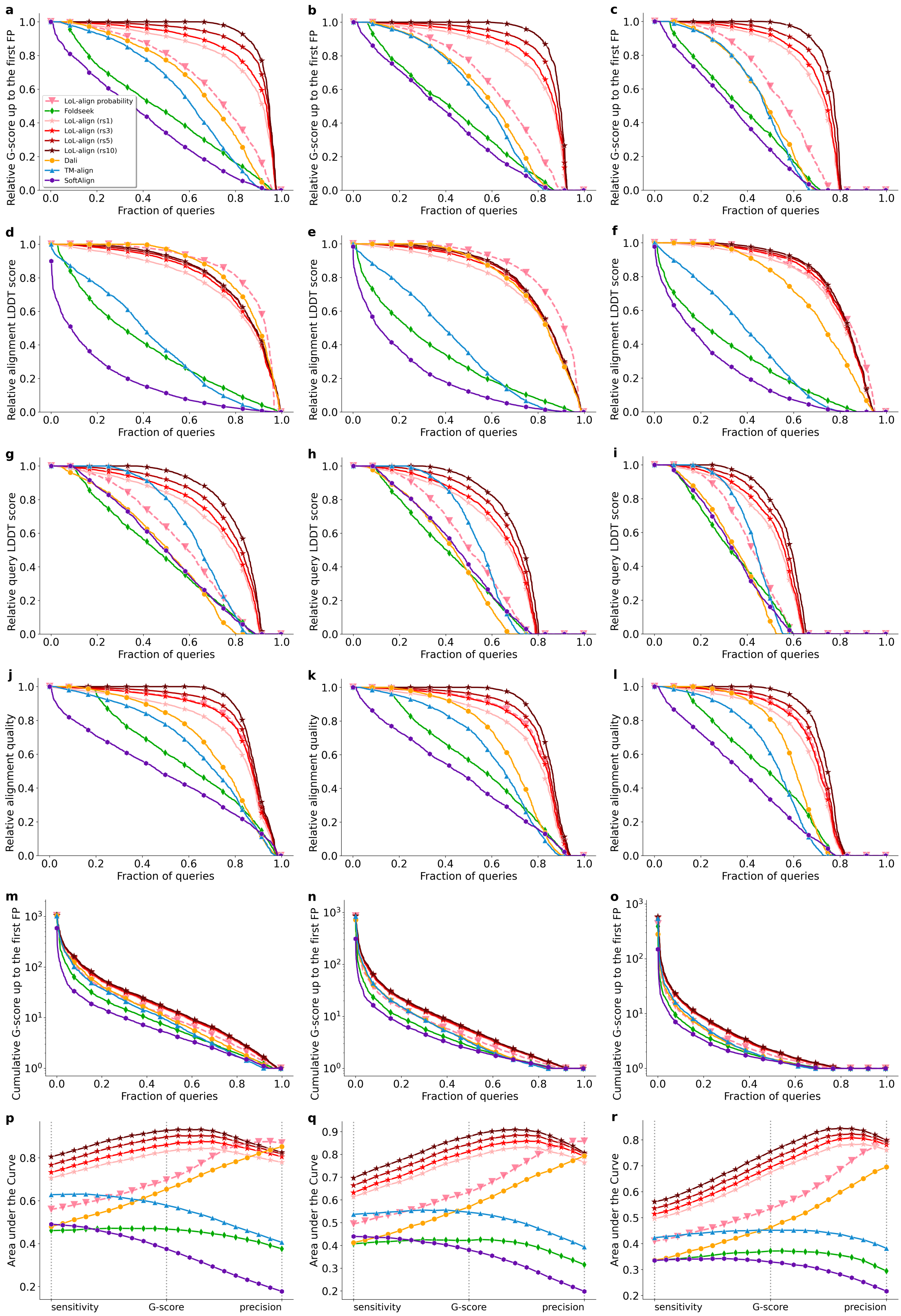

Figure 4: **Detailed analysis of the full-length structure alignment benchmark.** Each column corresponds to a different cutoff used to define true positives (0.4, 0.5, and 0.6 from left to right). The first row (**a–c**) shows the same benchmark as in Fig. 2**d**. The second row (**d–f**) displays results for the LDDT score normalized by alignment length (precision score). The third row (**g–i**) shows results for the LDDT score normalized by query length (sensitivity score). The fourth row (**j–l**) reproduces the analysis from Fig. 2**f**, the fifth row (**m–o**) is the cumulative up to the first FP and the sixed row (**p–r**) shows the same benchmark as in Fig. 2**e** . LoL-align (rs X) stands for the different refined seed that can be set with `--refine-seeds` X

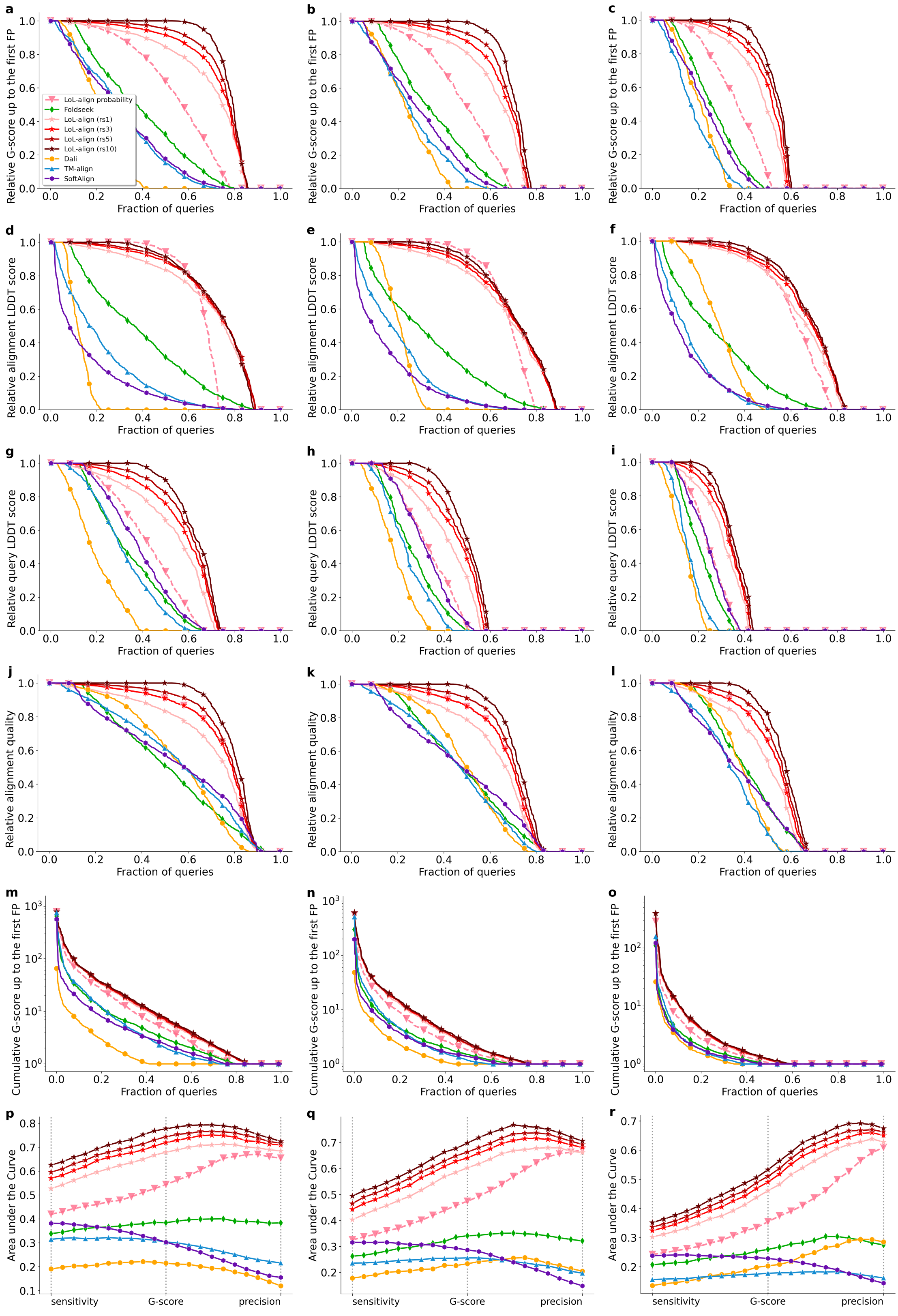

Figure 5: **Detailed analysis of the full-length structure alignment benchmark with low pLDDT regions.** Each column corresponds to a different cutoff used to define true positives (0.4, 0.5, and 0.6 from left to right). The first row (**a**–**c**) shows the same benchmark as in Fig. 2**d**. The second row (**d**–**f**) displays results for the LDDT score normalized by alignment length (precision score). The third row (**g**–**i**) shows results for the LDDT score normalized by query length (sensitivity score). The fourth row (**j**–**l**) reproduces the analysis from Fig. 2**f**, the fifth row (**m**–**o**) is the cumulative up to the first FP and the sixed row (**p**–**r**) shows the same benchmark as in Fig. 2**e** . LoL-align (rs X) stands for the different refined seed that can be set with `--refine-seeds` X

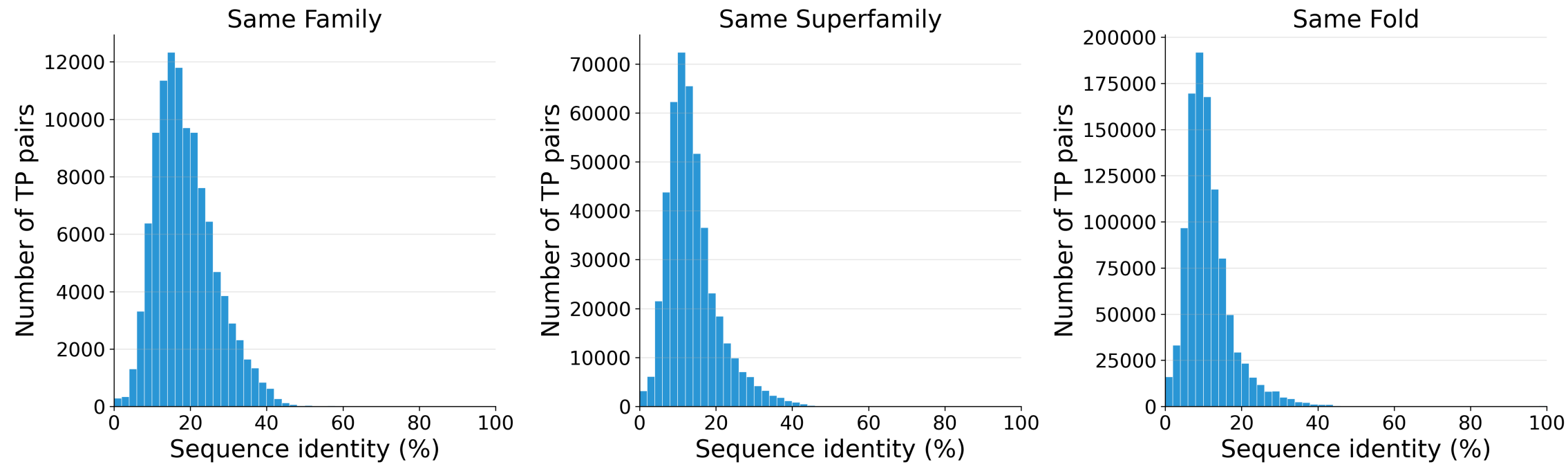

Figure 6: **LoL-align sequence identity distribution on SCOPe40** Distribution of pairwise sequence identity between true-positive structural homolog pairs in the SCOPe40 all-vs-all benchmark, stratified by SCOPe classification level: same family (left), same superfamily (centre) and same fold (right). Sequence identity is taken from the LoL-align pairwise alignment of each pair. Most true-positive pairs — especially at the superfamily and fold levels — fall in the low sequence identity regime.

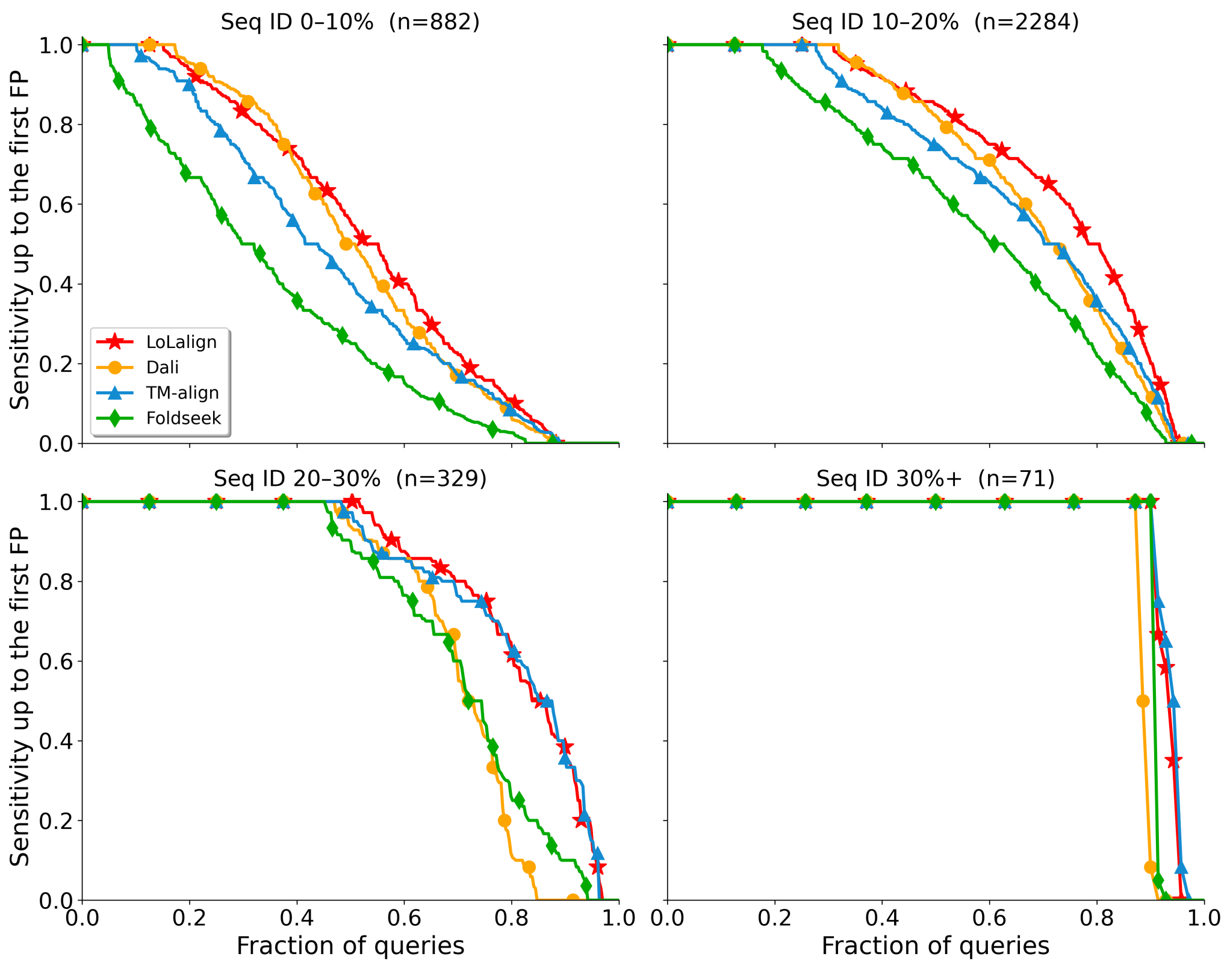

Figure 7: **Sequence Identity Benchmark.** ROC1 sensitivity of structural aligners stratified by mean pairwise sequence identity of true-positive superfamily hits. Each query in the SCOPe40 benchmark (n = 3,566) is assigned to one of four bins according to the mean sequence identity of its same-superfamily cross-family targets ($0-10\%, 10-20\%, 20-0\%, \geq 30\%$). Within each bin, curves show the per-query ROC1 sensitivity (fraction of superfamily true positives recovered before the first different fold false positive) sorted in descending order against the fraction of queries. n indicates the number of queries falling into the bin. The sequence identity is calculated based on LoL-align alignments.

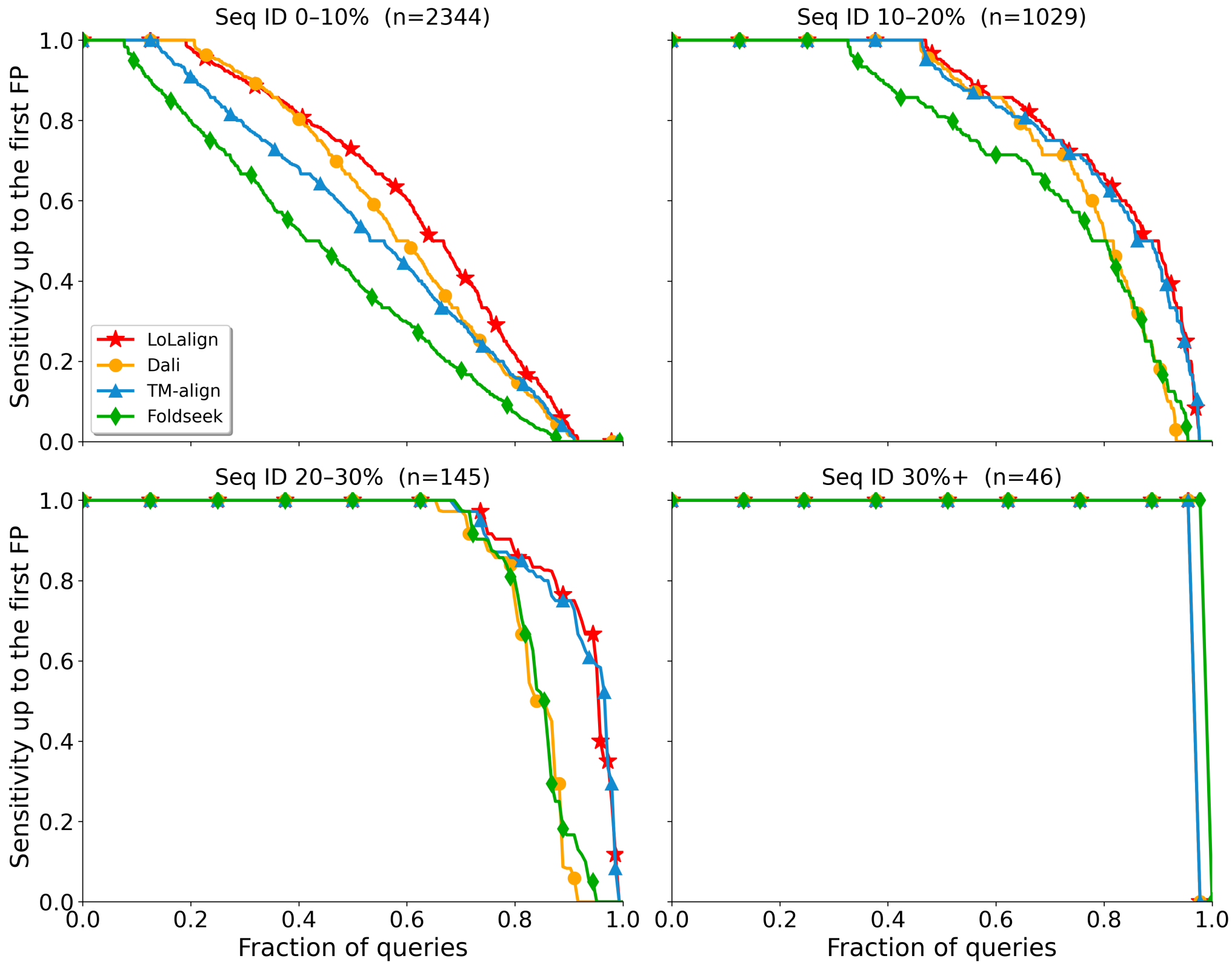

Figure 8: **Sequence Identity Benchmark.** ROC1 sensitivity of structural aligners stratified by mean pairwise sequence identity of true-positive superfamily hits. Each query in the SCOPe40 benchmark (n = 3,566) is assigned to one of four bins according to the mean sequence identity of its same-superfamily cross-family targets ($0-10\%, 10-20\%, 20-30\%, \geq 30\%$). Within each bin, curves show the per-query ROC1 sensitivity (fraction of superfamily true positives recovered before the first different-fold false positive) sorted in descending order against the fraction of queries. n indicates the number of queries falling into the bin. The sequence identity is calculated based on TM-align alignments.

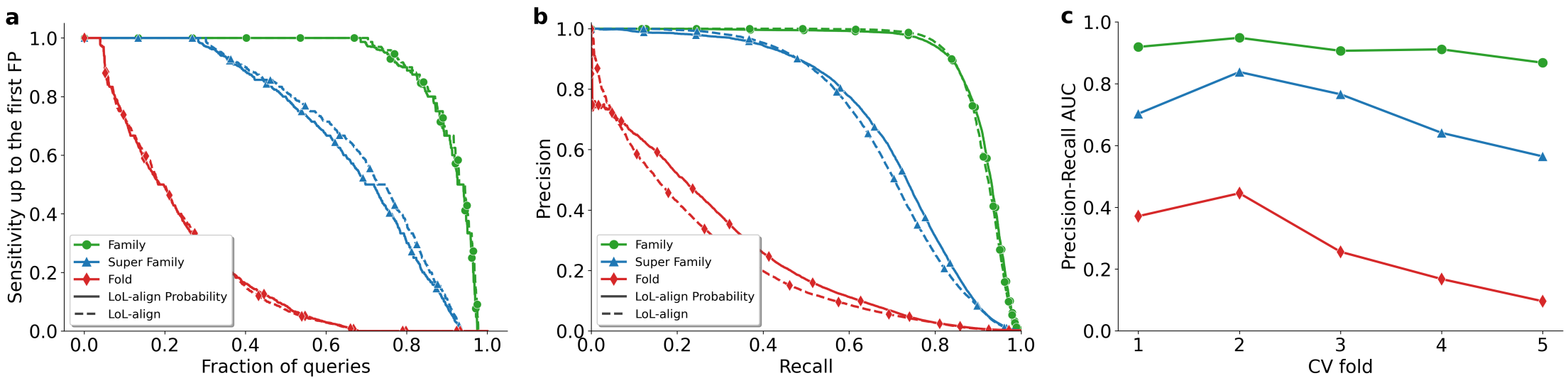

Figure 9: **Fold-disjoint cross-validation of the LoL-align probability classifier on SCOPe40.** A neural classifier ("LoL-align Probability") was trained in 5-fold cross-validation with folds split so that no SCOPe fold is shared between train and test. Each of the 3566 query domains is scored by the one model that did not train on it, and the held-out predictions are pooled into a single evaluation; metrics are reported at three SCOP levels — Family (green), Super Family (blue) and Fold (red). (a) ROC1: per-query sensitivity up to the first false positive, plotted against the fraction of queries (queries sorted by descending sensitivity). Solid lines, the LoL-align probability; dashed lines, the raw LoL-align score. (b) Precision–recall curves, solid/dashed convention as (c) Precision–recall AUC of the LoL-align probability for each of the five CV folds, showing fold-to-fold stability; performance decreases with increasing evolutionary distance (Family, Super Family , Fold) and varies more across folds at the harder levels.

Precision (%)	99.9	99	97	95	90	80	75	70	60	50
LoL score cutoff	0.699	0.649	0.619	0.596	0.560	0.516	0.5	0.488	0.466	0.445

Table 4: LoL-score cutoffs for Super Family-level precision.

	A	C	D	E	F	G	H	I	K	L	M	N	P	Q	R	S	T	V	W	Y
A	10	-1	1	7	6	2	2	-4	1	-1	-6	-2	-1	4	-1	-5	-1	-5	3	2
C	-1	7	-4	-5	-3	-2	-1	-10	-10	1	-11	1	-2	2	1	1	-6	1	-5	-5
D	1	-4	0	-3	-1	0	1	-4	-5	-6	-5	-4	-3	-3	-2	-6	-2	-6	-2	-2
E	7	-5	-3	15	3	-2	1	-8	-5	-4	-12	-5	-5	1	-3	-7	-5	-10	-2	3
F	6	-3	-1	3	10	1	1	-2	4	-1	-5	-3	-2	4	-2	-6	0	-6	7	0
G	2	-2	0	-2	1	10	7	3	-3	-5	3	1	-2	-1	5	-1	7	-5	-1	3
H	2	-1	1	1	1	7	10	0	-4	-4	-1	3	-1	0	4	-1	3	-4	-1	8
I	-4	-10	-4	-8	-2	3	0	11	-1	-10	11	-5	-6	-3	0	-7	9	-11	-2	-3
K	1	-10	-5	-5	4	-3	-4	-1	13	-9	-4	-9	-7	-2	-6	-12	-2	-13	9	-3
L	-1	1	-6	-4	-1	-5	-4	-10	-9	6	-13	-2	-3	3	-2	-3	-7	0	-6	-6
M	-6	-11	-5	-12	-5	3	-1	11	-4	-13	15	-6	-8	-7	-1	-8	7	-14	-2	-4
N	-2	1	-4	-5	-3	1	3	-5	-9	-2	-6	8	-1	-1	4	4	-2	0	-6	-1
P	-1	-2	-3	-5	-2	-2	-1	-6	-7	-3	-8	-1	1	-1	-1	-3	-4	-2	-5	-5
Q	4	2	-3	1	4	-1	0	-3	-2	3	-7	-1	-1	7	0	-2	-3	-1	1	-1
R	-1	1	-2	-3	-2	5	4	0	-6	-2	-1	4	-1	0	8	4	3	-1	-3	1
S	-5	1	-6	-7	-6	-1	-1	-7	-12	-3	-8	4	-3	-2	4	6	-4	0	-9	-5
T	-1	-6	-2	-5	0	7	3	9	-2	-7	7	-2	-4	-3	3	-4	11	-8	-2	0
V	-5	1	-6	-10	-6	-5	-4	-11	-13	0	-14	0	-2	-1	-1	0	-8	2	-9	-9
W	3	-5	-2	-2	7	-1	-1	-2	9	-6	-2	-6	-5	1	-3	-9	-2	-9	11	-2
Y	2	-5	-2	3	0	3	8	-3	-3	-6	-4	-1	-5	-1	1	-5	0	-9	-2	14

Table 5: Substitution scores for 3Di states. The scores are log-odds scores in half-bits, and were trained on Alphafold DB with TM-align alignments.
