# Supplementary material for: LoL-align: sensitive and fast probabilistic protein structure alignment
Source: Nat Methods. 2026 Oct 14;23(11):2147–50. doi: 10.1038/s41592-026-03249-w (PMC13736684; doi:10.1038/s41592-026-03249-w)
Supplement: Supplementary file 3 [file 41592_2026_3249_MOESM3_ESM.pdf]

nature portfolio

Peer Review File

LoL-align: sensitive and fast probabilistic protein structure alignment

Corresponding Author: Dr Johannes Soeding

This file contains all editorial decision letters in order by version, followed by all author rebuttals in order by version.

Version 0:

Decision Letter:

24th Feb 2026

Dear Johannes,

Your Brief Communication, "LoL-align: sensitive and fast probabilistic protein structure alignment", has now been seen by 2 reviewers. As you will see from their comments below, although the reviewers find your work of considerable potential interest, they have raised a number of concerns. We continue to be interested in potentially publishing your manuscript in Nature Methods, but would like to evaluate your response to these concerns before we reach a final decision on publication.

We therefore invite you to revise your manuscript to address these concerns.

We are committed to providing a fair and constructive peer-review process. Do not hesitate to contact us if there are specific requests from the reviewers that you believe are technically impossible or unlikely to yield a meaningful outcome.

When revising your paper:

* include a point-by-point response to the reviewers and to any editorial suggestions

* please underline/highlight any additions to the text or areas with other significant changes to facilitate review of the revised manuscript

* address the points listed described below to conform to our open science requirements

* ensure it complies with our general format requirements as set out in our guide to authors at www.nature.com/naturemethods

* resubmit all the necessary files by using the link below to access your home page

Link Redacted

Note: This URL links to your confidential account and associated information about manuscripts you may have submitted, or that you are reviewing for us. If you wish to forward this email to co-authors, please delete this link.

We hope to receive your revised paper within 6-8 weeks. If you are substantially delayed, please let us know. In this event, we will still be happy to reconsider your paper at a later date so long as nothing similar has been accepted for publication at Nature Methods or published elsewhere.

OPEN SCIENCE REQUIREMENTS

REPORTING SUMMARY

When revising your manuscript, please update your reporting summary.

Reporting summary: https://www.nature.com/documents/nr-reporting-summary.zip

If your paper includes custom software, we also ask you to complete a supplemental reporting summary.

Software supplement: https://www.nature.com/documents/nr-software-policy.pdf

Please submit these with your revised manuscript. They will be available to reviewers to aid in their evaluation if the paper is re-reviewed. If you have any questions about the checklist, please see http://www.nature.com/authors/policies/availability.html or contact me.

Please note that these forms are dynamic 'smart pdfs' and must therefore be downloaded and completed in Adobe Reader. We will then flatten them for ease of use by the reviewers. If you would like to reference the guidance text as you complete the template, please access these flattened versions at http://www.nature.com/authors/policies/availability.html.

For any revision that includes light microscopy data, we ask our authors to please include a completed light microscopy reporting table [https://www.nature.com/documents/Light_microscopy_reporting_table.xlsx] to ensure the methods are described thoroughly. The table will be available to reviewers and ultimately published should the manuscript be accepted at the journal.

EXTENDED DATA FIGURES
When re-submitting your manuscript, please ensure that any supplementary figures and tables that are crucial to the manuscript's conclusions are converted into Extended Data figures and tables to increase visibility of these data. Extended Data figures and tables are online-only (present in the online PDF and full-text HTML versions of the paper), peer-reviewed display items that provide essential background to the article but are not included in the main article due to space constraints. A maximum of ten Extended Data display items (figures and tables) is permitted.

DATA AVAILABILITY
We strongly encourage you to deposit all new data associated with the paper in a persistent repository where they can be freely and enduringly accessed. We recommend submitting the data to discipline-specific and community-recognized repositories; a list of repositories is provided here: http://www.nature.com/sdata/policies/repositories

All novel DNA and RNA sequencing data, protein sequences, genetic polymorphisms, linked genotype and phenotype data, gene expression data, macromolecular structures, and proteomics data must be deposited in a publicly accessible database, and accession codes and associated hyperlinks must be provided in the "Data Availability" section.

For papers containing bioimaging data, we strongly recommend depositing the data to Bioimage Archive (https://www.ebi.ac.uk/bioimage-archive/). Associated accession codes and hyperlinks should be provided in the "Data Availability" section.

Refer to our data policies here: https://www.nature.com/nature-research/editorial-policies/reporting-standards#availability-of-data

To further increase transparency, we encourage you to provide, in tabular form, the data underlying the graphical representations used in your figures. This is in addition to our data-deposition policy for specific types of experiments and large datasets. For readers, the source data will be made accessible directly from the figure legend. Spreadsheets can be submitted in .xls, .xlsx or .csv formats. Only one (1) file per figure is permitted: thus if there is a multi-paneled figure the source data for each panel should be clearly labeled in the csv/Excel file; alternately the data for a figure can be included in multiple, clearly labeled sheets in an Excel file. File sizes of up to 30 MB are permitted. When submitting source data files with your manuscript please select the Source Data file type and use the Title field in the File Description tab to indicate which figure the source data pertains to.

Please include a "Data availability" subsection in the Online Methods. This section should inform readers about the availability of the data used to support the conclusions of your study, including accession codes to public repositories, references to source data that may be published alongside the paper, unique identifiers such as URLs to data repository entries, or data set DOIs, and any other statement about data availability. At a minimum, you should include the following statement: "The data that support the findings of this study are available from the corresponding author upon request", describing which data is available upon request and mentioning any restrictions on availability. If DOIs are provided, please include these in the Reference list (authors, title, publisher (repository name), identifier, year). For more guidance on how to write this section please see: http://www.nature.com/authors/policies/data/data-availability-statements-data-citations.pdf

CODE AVAILABILITY
Please include a "Code Availability" subsection in the Online Methods which details how your custom code is made available. Only in rare cases (where code is not central to the main conclusions of the paper) is the statement "available upon request" allowed (and reasons should be specified).

We request that you deposit code in a DOI-minting repository such as Zenodo, Gigantum or Code Ocean and cite the DOI in the Reference list. We also request that you use code versioning and provide a license.

For more information on our code sharing policy and requirements, please see:
https://www.nature.com/nature-research/editorial-policies/reporting-standards#availability-of-computer-code

MATERIALS AVAILABILITY
As a condition of publication in Nature Methods, authors are required to make unique materials promptly available to others without undue qualifications.

Authors reporting new chemical compounds must provide chemical structure, synthesis and characterization details. Authors reporting mutant strains and cell lines are strongly encouraged to use established public repositories.

More details about our materials availability policy can be found at https://www.nature.com/nature-portfolio/editorial-policies/reporting-standards#availability-of-materials

ORCID
Nature Methods is committed to improving transparency in authorship. As part of our efforts in this direction, we are now requesting that all authors identified as 'corresponding author' on published papers create and link their Open Researcher and Contributor Identifier (ORCID) with their account on the Manuscript Tracking System (MTS), prior to acceptance. This applies to primary research papers only. ORCID helps the scientific community achieve unambiguous attribution of all scholarly contributions. You can create and link your ORCID from the home page of the MTS by clicking on 'Modify my Springer Nature account'. For more information please visit please visit www.springernature.com/orcid.

Please do not hesitate to contact me if you have any questions or would like to discuss these revisions further. We look forward to seeing your revised manuscript and thank you for the opportunity to consider your work.

Best regards,
Arunima

Arunima Singh, Ph.D.
Senior Editor
Nature Methods

Reviewers' Comments:

Reviewer #1 (Remarks to the Author):

This manuscript reports a highly sensitive and fast probabilistic tool (LoL-align) for protein structure alignment. The tool has the following major advantages or innovations:

Frist, it is not only more sensitive in identifying homologous proteins than other widely used tools for protein structure alignment including DALI and TM-align but also much faster than them (20 times faster than DALI and five times faster than TM-align). Moreover, the quality of alignments generated by LoL-align is substantially better than that of the other tools. Therefore, LoL-align is very useful for protein structure alignment and search, which has become indispensable for biological research as a vast amount of high-quality predicted protein structures are available nowadays.

Second, a Local-Log-odds scoring function used in LoL-align is novel. The novel score is a key reason that it achieves better sensitivity than other methods.

Third, a new vectorized Forward–Backward probabilistic sequence alignment methods that can leverage GPU computing architecture is developed, which makes it much faster than DALI and TM-align.

Fourth, the tool has been integrated with a widely used, super-fast protein structure database search tool – FoldSeek, making it much easier for users to use it. This can greatly increase the impact of the tool.

In addition to the significant advantages above, the experiment in this work is well designed. The results are rigorously evaluated.

Overall, LoL-align represents a very significant, much needed advance in the field of protein structure alignment and search

and is expected to have a strong impact on protein structure prediction, analysis, and usage.

Below are a few minor comments for the authors to consider:

(1) In this work, SCOPe40 (11,211 proteins clustered at 40% sequence identity from SCOPe 2.01), was used for evaluation. The query set comprised 3,566 structures, restricted to SCOPe entries with at least one additional family-, superfamily-, or foldlevel member. What is the distribution of the sequence identity between the proteins in the evaluation set (11,211 proteins) and those in the query set? How does LoL-align's performance change when the sequence identity between the proteins in the query set and the ones in the evaluation set changes from high to low? The answers to these questions can help users understand how well LoL-align works when the sequence similarity between query proteins and target proteins is low.

(2) Please discuss if it is possible to assign a statistical significance score (e.g., p-value) to the alignment score calculated by LoL-align.

(3) In this sentence "we selected 2,000 structures from the 2.3 million cluster representatives in the clustered AFDB and sampled 2000 query proteins with at least 100 residues scoring ≥ 0.7 predicted Local Distance Difference Test (pLDDT) score", are the 2000 structures and the sampled 2000 query proteins the same thing? Right now, they are described separately, making it read like the 2000 query proteins are sampled from the 2000 structures. If they refer to the same 2000 proteins, the sentence can be revised to reflect it. For instance, it can be revised as "We identified 2,000 structures from the AFDB's 2.3 million cluster representatives as query proteins, requiring each structure to have a minimum of 100 residues with a pLDDT score ≥ 0.7.".

Reviewer #1 (Remarks on code availability):

The code of the tool is released as a part of the widely used FoldSeek package at GitHub.

Reviewer #2 (Remarks to the Author):

This study introduces LoL-align, a novel protein structure alignment algorithm that leverages a Local Log-odds scoring function and a vectorized Forward-Backward algorithm to achieve a balance between sensitivity and computational speed. The benchmarking results on SCOPe and AFDB datasets demonstrate that LoL-align offers superior sensitivity for remote homologs compared to traditional tools like TM-align and Dali while being significantly faster. However, while the work is solid, several critical aspects regarding the algorithm's robustness, the scope of benchmarking, and its relationship with existing methodologies require further clarification and refinement.

1. The algorithm may be highly dependent on the initial "seed anchors." If the initial 7-mer seed is chosen incorrectly, will the iterative process fall into a local optimum or lead to erroneous alignments? I suggest that the authors supplement a sensitivity analysis on the impact of seed selection on the final alignment quality, particularly for proteins with numerous low-pLDDT regions.
2. The authors mention that deep learning methods struggle when processing proteins outside their training distribution; however, the benchmarks primarily compare LoL-align with traditional tools such as TM-align and Dali. In recent years, many structural alignment or search tools based on deep learning have emerged, such as TM-Vec and DeepBLAST. Comparing only with traditional algorithms may not fully demonstrate the competitiveness of LoL-align. It is recommended to supplement the performance comparison with representative deep learning alignment tools.
3. LoL-align emphasizes its sensitivity advantage in detecting remote homology. Given that FoldMason can capture more conserved evolutionary signals through multi-sequence information, which approach is more accurate for defining domain boundaries when dealing with extremely remote homology pairs: pairwise refinement using LoL-align or direct MSA construction using FoldMason? Do the authors have clear recommendations for the usage workflow? I believe providing these clarifications is crucial for further promoting the broad adoption of the tool.
4. On page 6, the LoL-score is trained on the alignment results of TM-align. Since the training labels originate from TM-align, is LoL-align essentially "learning and optimizing" the behavior of TM-align? The authors are encouraged to discuss how LoL-align achieves results that exceed (rather than merely approximate) its training baseline.
5. On Page 8, although the authors emphasize that the choice of 3Di weight (2.1) and AA weight (1.4) follows the methodological settings of Foldseek, is there a risk of overfitting these parameters to specific datasets used in that study, such as SCOPe? Evidence of generalizability to more diverse or recently predicted structural databases would be beneficial.

Reviewer #2 (Remarks on code availability):

Good

Version 1:

Decision Letter:

Our ref: NMETH-BC63737A

16th Jul 2026

Dear Dr. Soeding,

Thank you for submitting your revised manuscript "LoL-align: sensitive and fast probabilistic protein structure alignment" (NMETH-BC63737A). It has now been seen by the original referees and their comments are below. The reviewers find that the paper has improved in revision, and therefore we'll be happy in principle to publish it in Nature Methods, pending minor revisions to satisfy the referees' final requests and to comply with our editorial and formatting guidelines.

We are now performing detailed checks on your paper and will send you a checklist detailing our editorial and formatting requirements within two weeks or so. Please do not upload the final materials and make any revisions until you receive this additional information from us.

TRANSPARENT PEER REVIEW
Nature Methods offers a transparent peer review option for new original research manuscripts submitted from 17th February 2021. We encourage increased transparency in peer review by publishing the reviewer comments, author rebuttal letters and editorial decision letters if the authors agree. Such peer review material is made available as a supplementary peer review file. **Please state in the cover letter 'I wish to participate in transparent peer review' if you want to opt in, or 'I do not wish to participate in transparent peer review' if you don't.** Failure to state your preference will result in delays in accepting your manuscript for publication.

Please note: we allow redactions to authors' rebuttal and reviewer comments in the interest of confidentiality. If you are concerned about the release of confidential data, please let us know specifically what information you would like to have removed. Please note that we cannot incorporate redactions for any other reasons. Reviewer names will be published in the peer review files if the reviewer signed the comments to authors, or if reviewers explicitly agree to release their name. For more information, please refer to our FAQ page.

ORCID
IMPORTANT: Non-corresponding authors do not have to link their ORCIDs but are encouraged to do so. Please note that it will not be possible to add/modify ORCIDs at proof. Thus, please let your co-authors know that if they wish to have their ORCID added to the paper they must follow the procedure described in the following link prior to acceptance: https://www.springernature.com/gp/researchers/orcid/orcid-for-nature-research

Author names using non-Roman characters
Nature Portfolio journals can support presentation of author names using non-Roman characters in the HTML version of the article. If you wish to, please include author names in parentheses after the Roman-character spelling; see example online here. Currently supported scripts are: Arabic, Chinese, Cyrillic, Devanagari, Greek, Hebrew, Hangul, Japanese and Persian. You will be asked to verify the rendering is correct at proof stage.

Thank you again for your interest in Nature Methods. Please do not hesitate to contact me if you have any questions. We will be in touch again soon.

Sincerely,
Arunima

Arunima Singh, Ph.D.
Senior Editor
Nature Methods

Reviewer #1 (Remarks to the Author):

The revised manuscript addressed all my previous comments satisfactorily. The method is a significant contribution to protein structure alignment and can have broad application in the field.

Reviewer #2 (Remarks to the Author):

I thank the authors for their careful and constructive revision. In the revised manuscript and response letter, the authors have adequately addressed my previous concerns. In particular, the additional clarifications, analyses, and revisions have improved the rigor and transparency of the study. I am satisfied with the authors' responses and do not have further major concerns.

Reviewer #2 (Remarks on code availability):

I have reviewed the code-related materials.

INTERNAL USE ONLY AIP modules required: ["Life Science"]

Version 2:

Decision Letter:

8th Sep 2026

Dear Johannes,

I am pleased to inform you that your Brief Communication, "LoL-align: sensitive and fast probabilistic protein structure alignment", has now been accepted for publication in Nature Methods. The received and accepted dates will be November 24, 2025 and September 8, 2026. This note is intended to let you know what to expect from us over the next month or so, and to let you know where to address any further questions.

Over the next few weeks, your paper will be copyedited to ensure that it conforms to Nature Methods style. Once your paper is typeset, you will receive an email with a link to choose the appropriate publishing options for your paper and our Author Services team will be in touch regarding any additional information that may be required.

If you have any questions about our publishing options, costs, Open Access requirements, or our legal forms, please contact ASJournals@springernature.com

Once proofs are generated, they will be sent to you electronically and you will be asked to send a corrected version within 48 hours. It is extremely important that you let us know now whether you will be difficult to contact over the next month. If this is the case, we ask that you send us the contact information (email, phone and fax) of someone who will be able to check the proofs and deal with any last-minute problems.

If, when you receive your proof, you cannot meet the deadline, please inform us at rjsproduction@springernature.com immediately.

If you have posted a preprint on any preprint server, please ensure that the preprint details are updated with a publication reference, including the DOI and a URL to the published version of the article on the journal website.

You may wish to make your media relations office aware of your accepted publication, in case they consider it appropriate to organize some internal or external publicity. Once your paper has been scheduled you will receive an email confirming the publication details. This is normally 3-4 working days in advance of publication. If you need additional notice of the date and time of publication, please let the production team know when you receive the proof of your article to ensure there is sufficient time to coordinate. Further information on our embargo policies can be found here: https://www.nature.com/authors/policies/embargo.html

Authors may need to take specific actions to achieve compliance with funder and institutional open access mandates. If your research is supported by a funder that requires immediate open access (e.g. according to Plan S principles) then you should select the gold OA route, and we will direct you to the compliant route where possible. For authors selecting the subscription publication route, the journal's standard licensing terms will need to be accepted, including self-archiving policies. Those licensing terms will supersede any other terms that the author or any third party may assert apply to any version of the manuscript.

If you are active on X or Bluesky, please e-mail me your and your coauthors' handles so that we may tag you when the paper is published.

To assist our authors in disseminating their research to the broader community, our SharedIt initiative provides you with a unique shareable link that will allow anyone (with or without a subscription) to read the published article. Recipients of the link with a subscription will also be able to download and print the PDF. As soon as your article is published, you will receive an automated email with your shareable link.

Please note that you and your coauthors may order reprints and single copies of the issue containing your article through Springer Nature Limited's reprint website, which is located at http://www.nature.com/reprints/author-reprints.html. If there are any questions about reprints please send an email to author-reprints@nature.com and someone will assist you.

You can now use a single sign-on for all your accounts, view the status of all your manuscript submissions and reviews, access usage statistics for your published articles and download a record of your refereeing activity for the Nature journals.

Please feel free to contact me if you have questions about any of these points.

Best regards,
Arunima

Arunima Singh, Ph.D.
Senior Editor
Nature Methods

** Visit the Springer Nature Editorial and Publishing website at www.springernature.com/editorial-and-publishing-jobs for more information about our career opportunities. If you have any questions please click here.**

Open Access This Peer Review File is licensed under a Creative Commons Attribution 4.0 International License, which permits use, sharing, adaptation, distribution and reproduction in any medium or format, as long as you give appropriate credit to the original author(s) and the source, provide a link to the Creative Commons license, and indicate if changes were made. In cases where reviewers are anonymous, credit should be given to 'Anonymous Referee' and the source.
The images or other third party material in this Peer Review File are included in the article's Creative Commons license, unless indicated otherwise in a credit line to the material. If material is not included in the article's Creative Commons license and your intended use is not permitted by statutory regulation or exceeds the permitted use, you will need to obtain permission directly from the copyright holder.
To view a copy of this license, visit https://creativecommons.org/licenses/by/4.0/

Reviewer #1

This manuscript reports a highly sensitive and fast probabilistic tool (LoL-align) for protein structure alignment. The tool has the following major advantages or innovations:

First, it is not only more sensitive in identifying homologous proteins than other widely used tools for protein structure alignment, including DALI and TM-align, but also much faster than them (20 times faster than DALI and five times faster than TM-align). Moreover, the quality of alignments generated by LoL-align is substantially better than that of the other tools. Therefore, LoL-align is very useful for protein structure alignment and search, which has become indispensable for biological research as a vast amount of high-quality predicted protein structures are available nowadays.

Second, a Local-Log-odds scoring function used in LoL-align is novel. The novel score is a key reason that it achieves better sensitivity than other methods.

Third, a new vectorized Forward–Backward probabilistic sequence alignment method that can leverage GPU computing architecture is developed, which makes it much faster than DALI and TM-align.

Fourth, the tool has been integrated with a widely used, super-fast protein structure database search tool – FoldSeek, making it much easier for users to use it. This can greatly increase the impact of the tool.

In addition to the significant advantages above, the experiment in this work is well designed. The results are rigorously evaluated.

Overall, LoL-align represents a very significant, much-needed advance in the field of protein structure alignment and search and is expected to have a strong impact on protein structure prediction, analysis, and usage.

Below are a few minor comments for the authors to consider:
(1) In this work, SCOPe40 (11,211 proteins clustered at 40% sequence identity from SCOPe 2.01) was used for evaluation. The query set comprised 3,566 structures, restricted to SCOPe entries with at least one additional family-, superfamily-, or fold-level member. What is the distribution of the sequence identity between the proteins in the evaluation set (11,211 proteins) and those in the query set?

How does LoL-align's performance change when the sequence identity between the proteins in the query set and the ones in the evaluation set changes from high to low? The answers to these questions can help users understand how well LoL-align works when the sequence similarity between query proteins and target proteins is low.

We thank the reviewer for this insightful suggestion. To address it, we (i) computed the distribution of pairwise sequence identities between each query and its true-positive targets at the family, superfamily, and fold levels, and (ii) stratified the per-query ROC1 sensitivity by

the mean sequence identity of each query's same-superfamily cross-family targets. Sequence identities are derived from the structural alignments themselves; we report both LoL-align-based and TM-align-based binnings to ensure the analysis is not biased toward any single method.

The distributions confirm that SCOPe40 is dominated by remote homologs: at the superfamily level, the distribution peaks around 10–15% identity and decays rapidly above 25%, with essentially no pairs exceeding 40%. The fold-level distribution is shifted even further toward low identity, peaking around 8–12%. As expected, family-level pairs are slightly more similar but still concentrated in the twilight zone (peak ~15–20%). Aggregated into bins, the LoL-align-based binning yields n = 882, 2,284, 329, and 71 queries for the 0–10%, 10–20%, 20–30%, and ≥30% bins, respectively; the TM-align-based binning gives n = 2,344, 1,029, 145, and 46.

We added three plots to the Supplementary (S6-S8) and referred to them in the text as: “We also analyzed the performance on different sequence identity levels, see Figures. S6-S8”

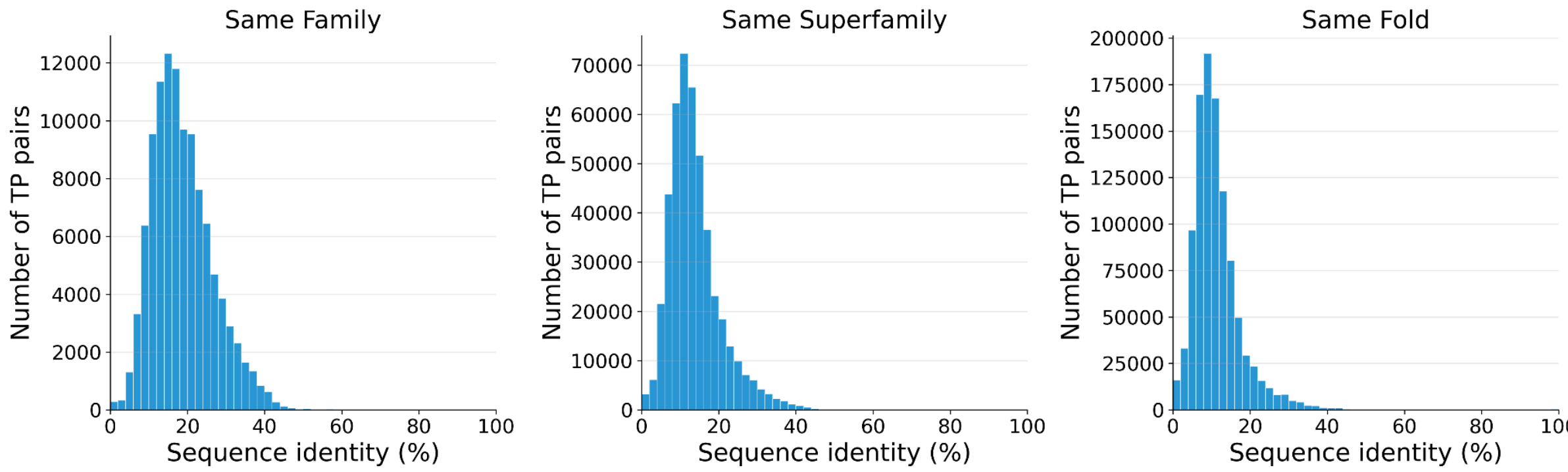

LoL-align Binning:

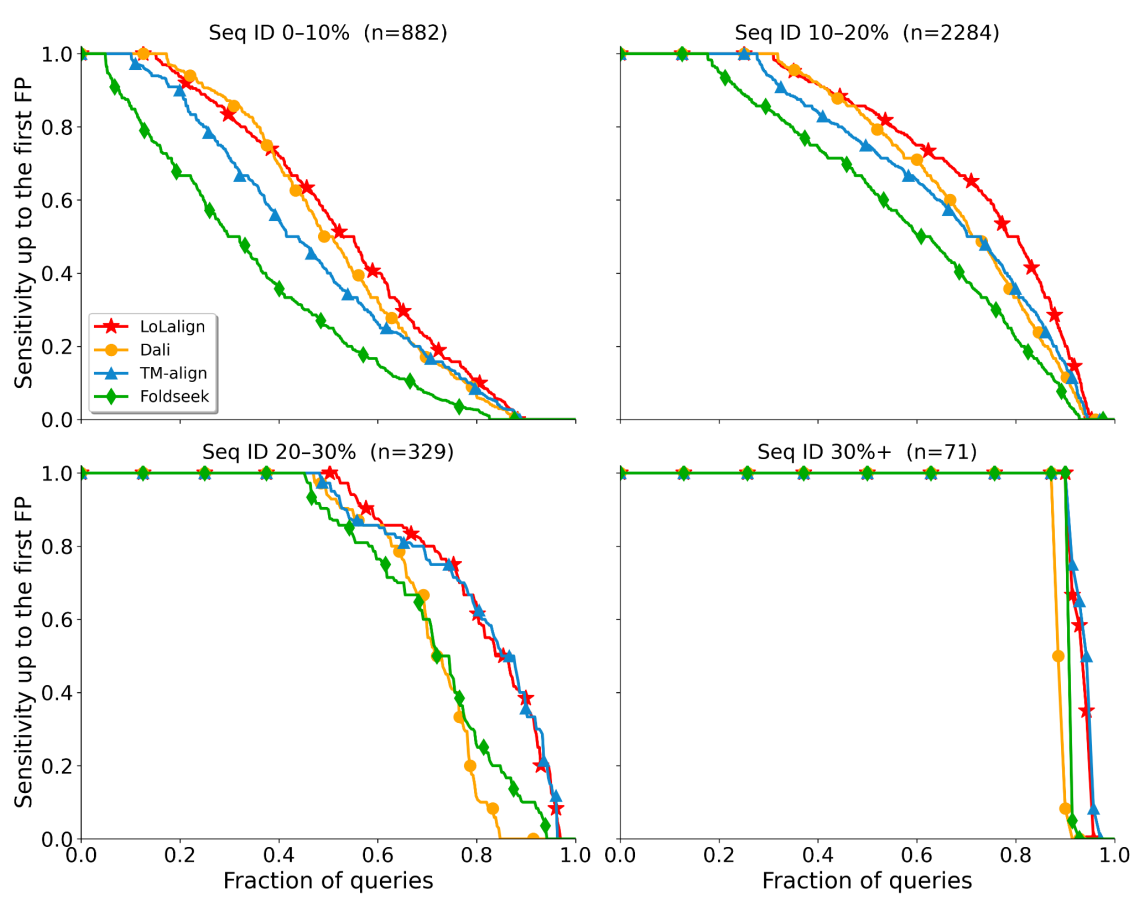

TM-align Binning:

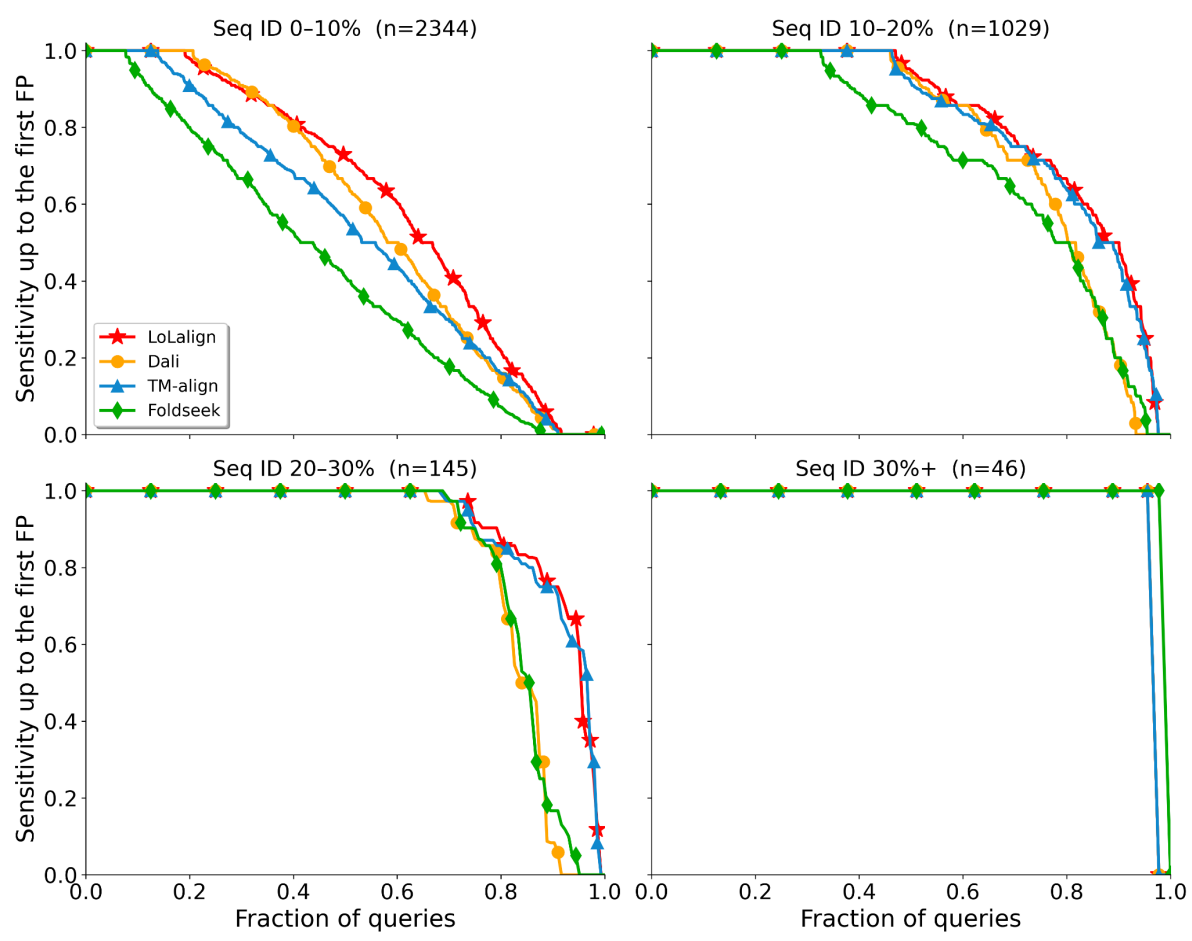

(2)Please discuss if it is possible to assign a statistical significance score (e.g., p-value) to the alignment score calculated by LoL-align.

We thank the reviewer for this insightful suggestion. Providing statistical significance estimates (such as e-values or p-values) is indeed a highly valuable feature for ranking and interpreting hits, particularly in large-scale structural database searches.

In principle, an e-value can be assigned to LoL-align scores by fitting an Extreme Value Distribution (EVD) to a background distribution of unrelated structure pairs—factoring in query and target lengths—as is standard practice in sequence and structure search tools like BLAST and Foldseek. While this is straightforward on curated datasets like SCOPe (where cross-fold pairs provide a clean, ground-truth negative set), doing so for full-length proteins in databases like the AlphaFold Database (AFDB) presents a major bottleneck. Without manual annotations, partitioning full-length structures into "related" and "unrelated" creates a circular dependency on the structural aligner itself, and an EVD fitted on single-domain SCOPe structures does not generalize well to multi-domain, full-length data.

To address this, we extensively explored methods to generate a reliable full-length null model. In sequence alignment, a common strategy for E-value calibration is database reversal. To translate this concept to structure space, we attempted to generate physical decoy structures by reversing C-alpha trajectories and reconstructing the backbones using a variety of methods—ranging from classical tools like PULCHRA to advanced deep-learning approaches like AlphaFold’s initial guess and Cg2all. We then evaluated whether these reconstructed decoys could replicate the empirical false-positive (cross-fold) distribution on SCOPe. Unfortunately, the reversed structures consistently failed to model the true background distribution, performing significantly worse than the original cross-fold negatives. To our knowledge, a robust method for generating a realistic null model for full-length structural data remains an open challenge in the field.

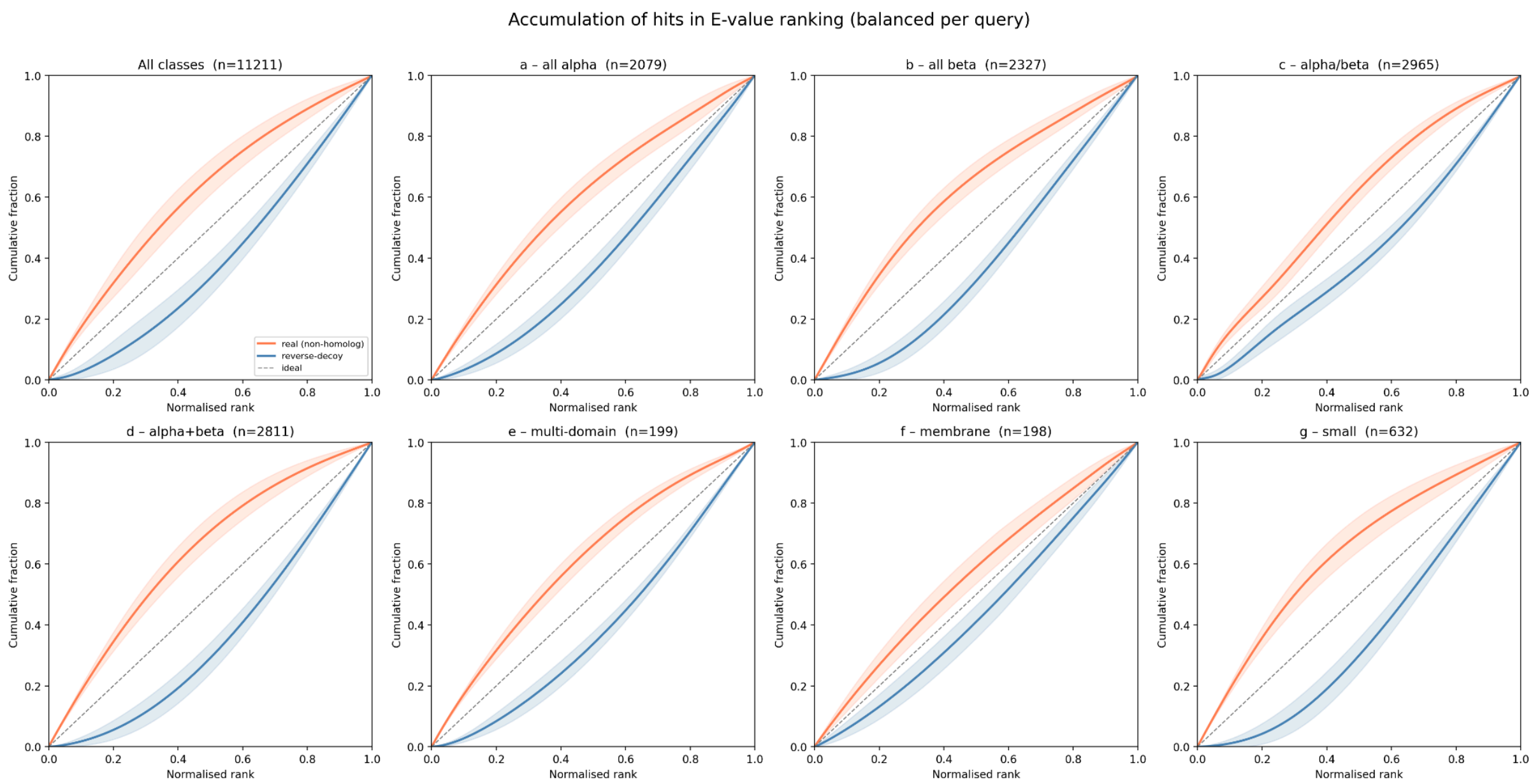

However, we entirely agree with the reviewer that an interpretable metric is necessary to help users distinguish true homologs from non-homologs. To solve this, we have introduced a lightweight neural network that predicts a homology probability ("LoL-align probability") based solely on local alignment features. We trained this model on SCOPe, defining superfamily members as true positives and cross-fold pairs as false positives. By strictly restricting the inputs to local structural features, the network can bypass global length artifacts and generalize well to full-length proteins.

We have rerun our full-length benchmark using this new probability score and observed an interesting trade-off: while the standard LoL-align score prioritizes global structural similarity (yielding higher sensitivity), the LoL-align probability score prioritizes shorter, high-quality local alignments, boosting precision on the benchmark. We believe this addition directly addresses the reviewer's core concern by providing users with an easily interpretable "probability of homology".

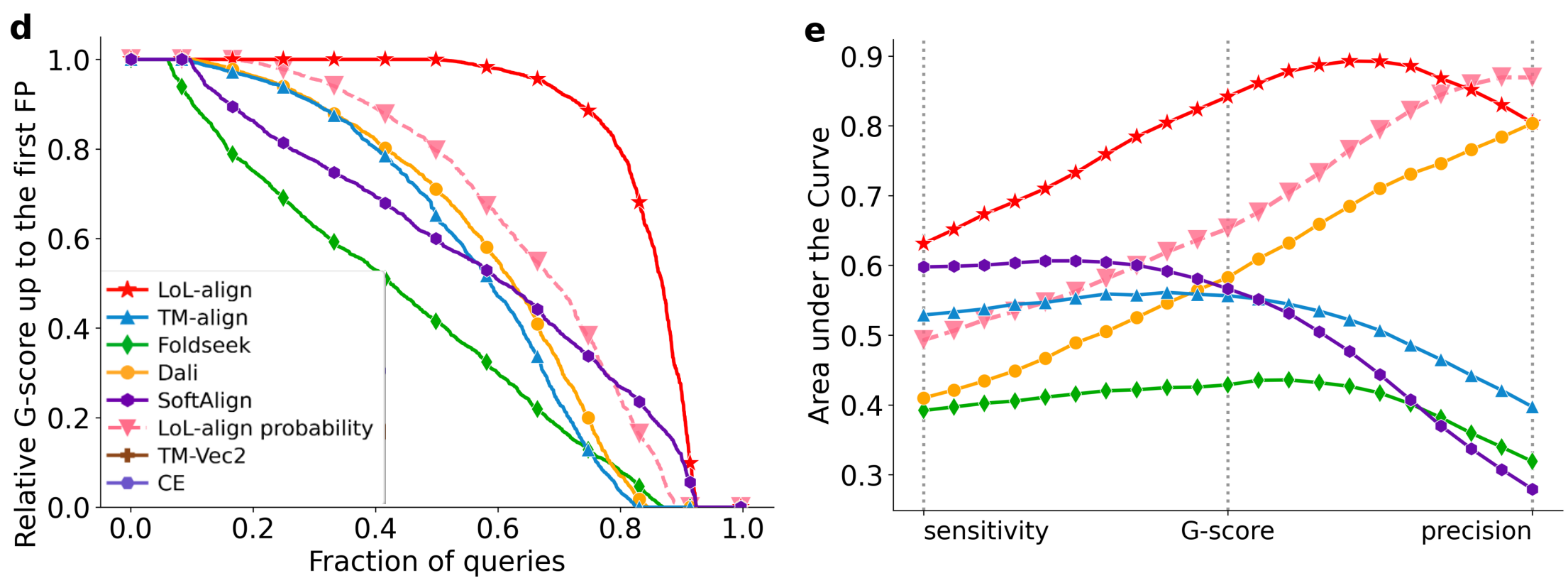

We added a new section in the Online method part, where we describe features, training, etc., and added a Cross-Validation benchmark in the Supplementary (S9).

We also mention the new Score in the main text as follows:

While the LoL-score reflects global similarity of two aligned proteins, it is less suited for determining whether a local alignment represents true homology. We therefore trained a small neural network on the SCOPe database to predict the probability of a homologous match from only local alignment features. On the single-domain benchmark, the probability performs similarly to the LoL-score (Fig. S9), while on the full-length benchmark, it places more emphasis on residue-wise precision than sensitivity (Fig 2d,e), Fig. S4.

(3) In this sentence, "we selected 2,000 structures from the 2.3 million cluster representatives
in the clustered AFDB and sampled 2000 query proteins with at least 100 residues scoring ≥ 0.7 predicted Local Distance Difference Test (pLDDT) score", are the 2000 structures and the sampled 2000 query proteins the same thing? Right now, they are described separately, making it read like the 2000 query proteins are sampled from the 2000 structures. If they refer to the same 2000 proteins, the sentence can be revised to reflect it. For instance, it can

be revised as "We identified 2,000 structures from the AFDB's 2.3 million cluster representatives as query proteins, requiring each structure to have a minimum of 100 residues with a pLDDT score ≥ 0.7.".

We thank the reviewer for pointing out this ambiguity. The reviewer is correct that the 2,000 structures and the 2,000 query proteins refer to the same set. We have revised the sentence in the manuscript to make this explicit.

Reviewer #2

This study introduces LoL-align, a novel protein structure alignment algorithm that leverages a Local Log-odds scoring function and a vectorized Forward-Backward algorithm to achieve a balance between sensitivity and computational speed. The benchmarking results on SCOPe and AFDB datasets demonstrate that LoL-align offers superior sensitivity for remote homologs compared to traditional tools like TM-align and Dali while being significantly faster. However, while the work is solid, several critical aspects regarding the algorithm's robustness, the scope of benchmarking, and its relationship with existing methodologies require further clarification and refinement.

1. The algorithm may be highly dependent on the initial "seed anchors." If the initial 7-mer seed is chosen incorrectly, will the iterative process fall into a local optimum or lead to erroneous alignments? I suggest that the authors supplement a sensitivity analysis on the impact of seed selection on the final alignment quality, particularly for proteins with numerous low-pLDDT regions.

We thank the reviewer for raising this important point. To address it, we have made the seed-selection step user-configurable by exposing two parameters: `--candidate-seeds`, the number of candidate seeds for which an ungapped structural alignment is computed, and `--refine-seeds`, the number of top-ranked seeds that are subsequently refined. This allows the trade-off between robustness against suboptimal seeds and runtime to be tuned explicitly.

To quantify the sensitivity to seed selection, we evaluated `--refine-seeds` values of 1, 3, 5, and 10 on both the SCOPe all-vs-all benchmark and the full-length AlphaFold benchmark, which includes proteins with extensive low-pLDDT regions. On SCOPe, performance increases monotonically with the number of refined seeds and begins to saturate around 5–10 seeds. On the full-length benchmark, increasing the number of refined seeds yields consistent improvements across all evaluated

metrics — G-score, LDDT normalized by alignment length (precision), LDDT normalized by query length (sensitivity), and overall alignment quality — for proteins with both high- and low-pLDDT regions.

We describe these features in the Online Method section as follows

“Because the final alignment depends on the selected anchors, we expose two user-configurable parameters: the number of candidate seeds for which an ungapped structure alignment is computed --candidate-seeds and the number subsequently refined top-ranked seeds --refine-seeds. The effect of --refine-seeds is reported in Table S2 and Figures S4 and S5.”

The New Low plddt benchmark is mentioned as follows:

“To evaluate performance on low-confidence regions, we additionally sampled 1000 queries containing at least 100 residues with pLDDT less than or equal to 0.5. Results for the high- and low-confidence sets are reported in Figures S4 and S5, respectively.”

In addition, to further reduce the risk of selecting spurious seeds in unstructured regions, we had re-fitted the 3Di substitution matrix on TM-align alignments from the AlphaFold database. The re-fitted matrix assigns lower scores to matches in unstructured regions than the original SCOPe-trained matrix, making low-pLDDT segments less likely to be chosen as initial anchors.

The new matrix is described in the Online Method part like this, and the Substitution matrix is shown in the supplementary:

“Furthermore, since the original 3Di substitution matrix was fitted on SCOPe and assigns high scores to matches in unstructured regions, we instead use a 3Di matrix re-fitted on TM-align alignments from the AlphaFold database, which yields lower scores for such spurious matches (Table S4)”

High pLDDT:

Each column has a different true positive cutoff. Left 0.4, middle 0.5, right 0.6.

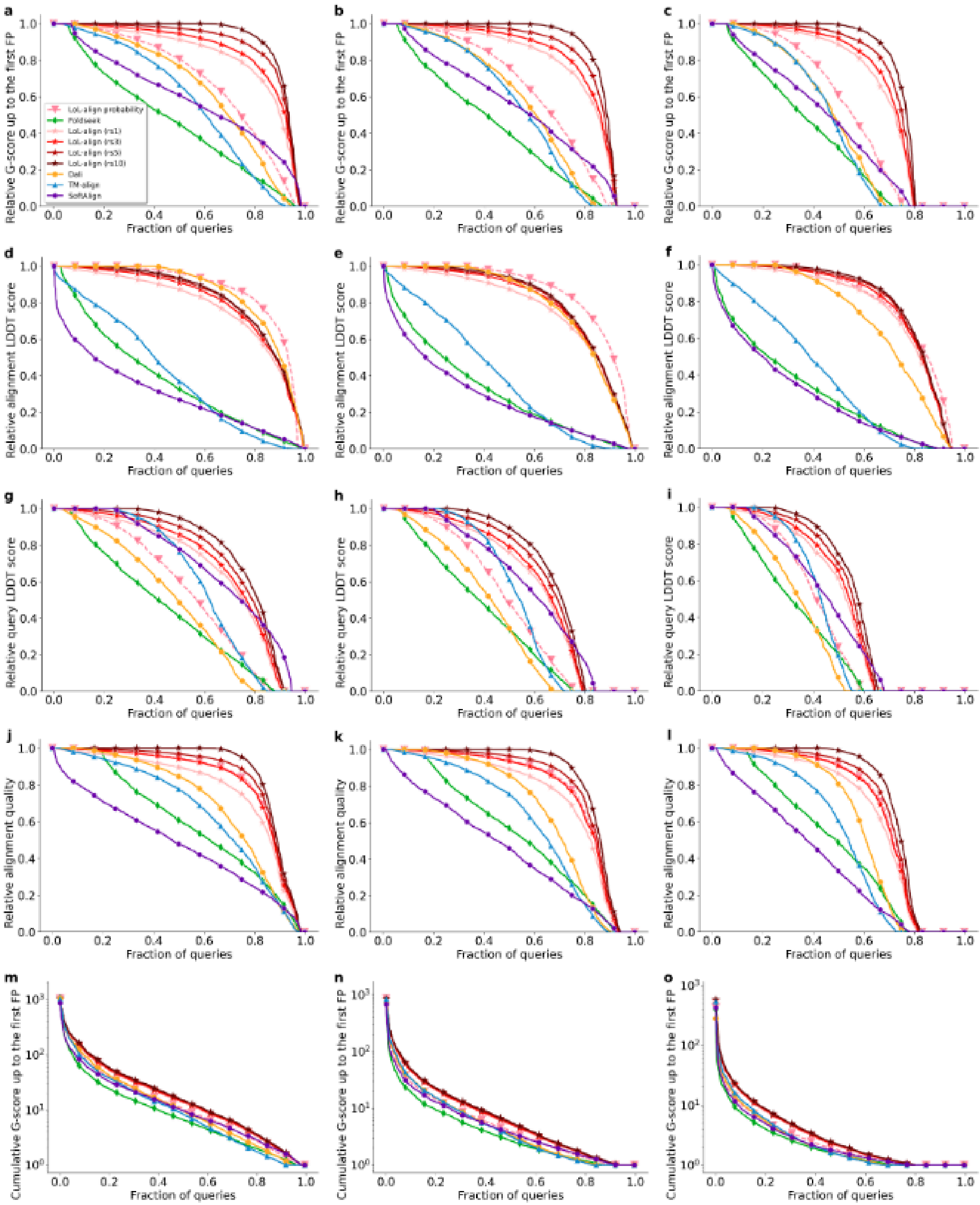

Low pLDDT:

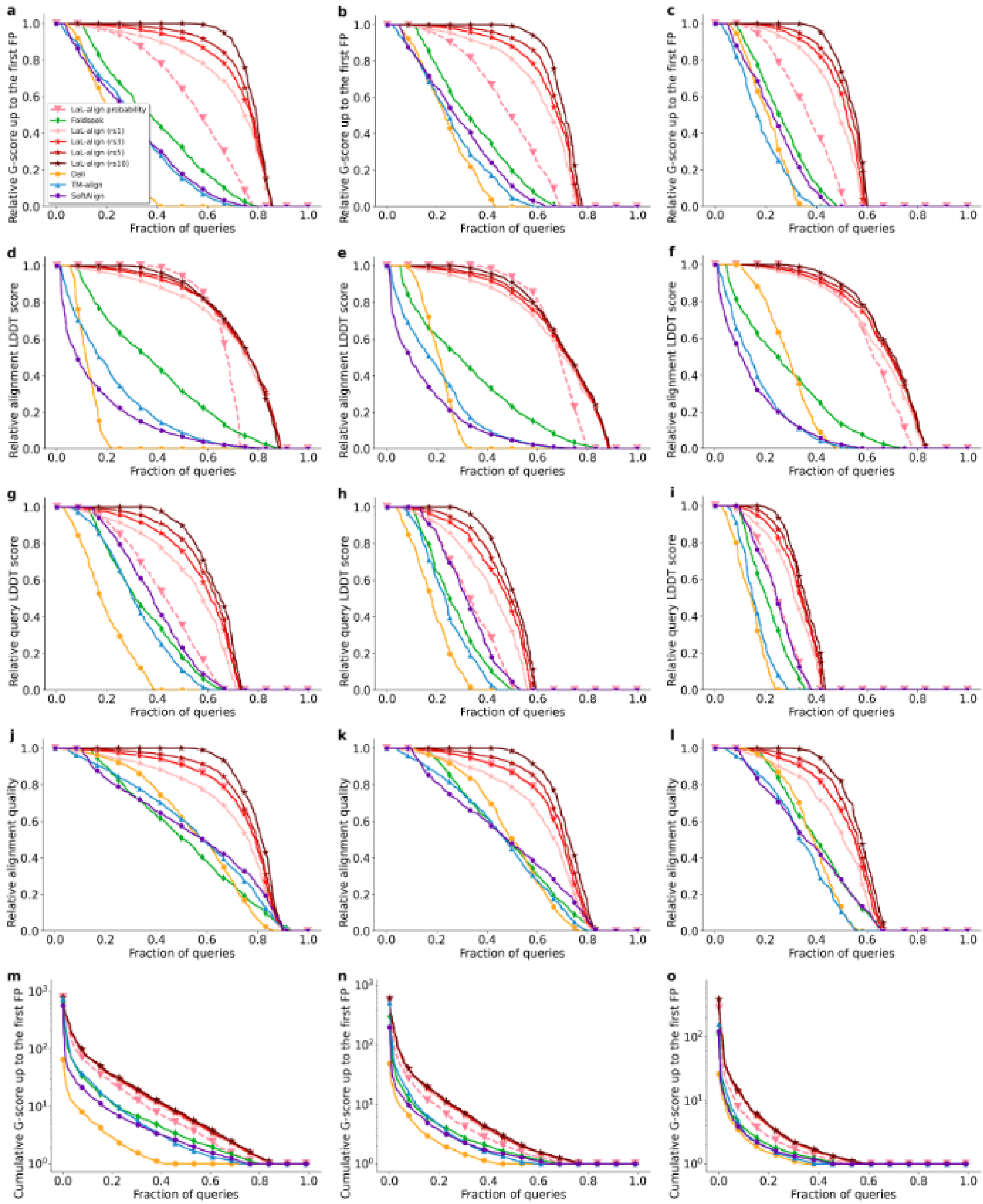
a
b
c
Relative G-score up to the first FP
Fraction of queries
LoL-align probability
Foldseek
LoL-align (rs1)
LoL-align (rs3)
LoL-align (rs5)
LoL-align (rs10)
Dali
TM-align
SoftAlign
d
e
f
Relative alignment LDDT score
g
h
i
Relative query LDDT score
j
k
l
Relative alignment quality
m
n
o
Cumulative G-score up to the first FP

2. The authors mention that deep learning methods struggle when processing proteins outside their training distribution; however, the benchmarks primarily compare LoL-align with traditional tools such as TM-align and Dali. In recent years, many structural alignment or search tools based on deep learning have emerged, such as TM-Vec and DeepBLAST. Comparing only with traditional algorithms may not fully demonstrate the competitiveness of LoL-align. It is recommended to supplement the performance comparison with representative deep learning alignment tools.

We thank the reviewer for this suggestion. We have added two recent deep-learning-based methods to our benchmarks: **SoftAlign**, a neural structural aligner, and **TM-Vec2**, a sequence-based structural search method.

SoftAlign has been added to the full-length AlphaFold benchmarks alongside the existing baselines. It is worth mentioning that Soft-align uses a soft LDDT score to rank the alignments by default, which can boost the performance on this benchmark

TM-Vec2 operates on sequence input only and produces per-structure embeddings for retrieval rather than residue-level alignments. We therefore included it only in the SCOPe all-vs-all benchmark, where retrieval performance can be compared directly. LoL-align achieves higher sensitivity than TM-Vec2, particularly at the superfamily and fold levels, consistent with the expectation that structure-based aligners retain an advantage over sequence-only methods in the remote-homology regime that dominates SCOPe40.

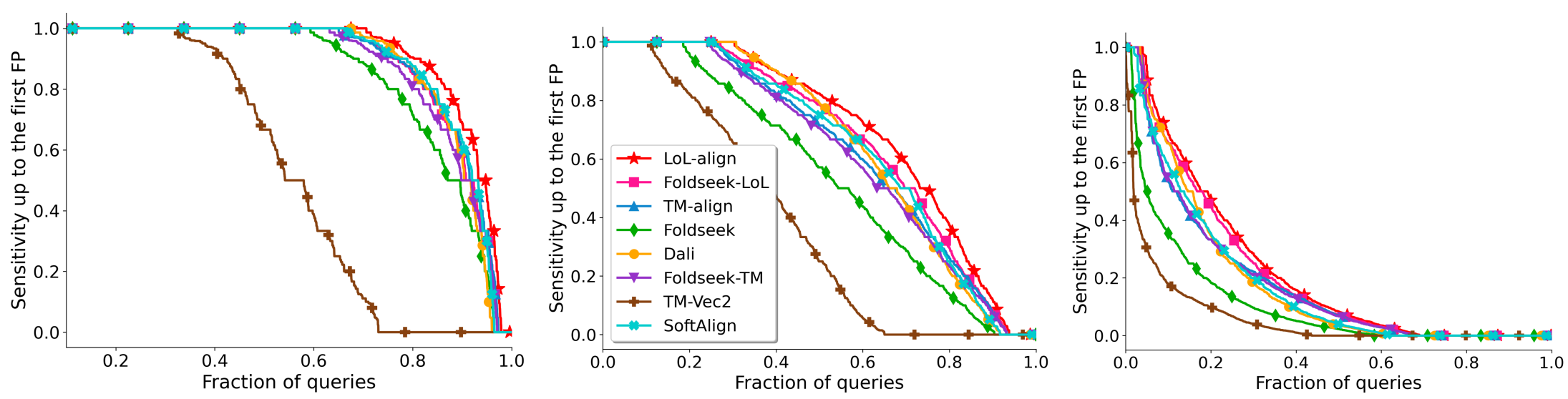

3. LoL-align emphasizes its sensitivity advantage in detecting remote homology. Given that FoldMason can capture more conserved evolutionary signals through multi-sequence information, which approach is more accurate for defining domain boundaries when dealing with extremely remote homology pairs: pairwise refinement using LoL-align or direct MSA construction using FoldMason? Do the authors have clear recommendations for the usage workflow? I believe providing these clarifications is crucial for further promoting the broad adoption of the tool.

We thank the reviewer for this insightful question, which touches on an important methodological distinction.

LoL-align and FoldMason address related but distinct problems. LoL-align is a *pairwise* structural aligner optimized for sensitivity in the remote-homology regime, while FoldMason is a *multiple* structure aligner that leverages information across many homologs simultaneously, which can indeed provide a stronger evolutionary signal when sufficiently many related structures are available. The two approaches are therefore complementary rather than alternative options.

Importantly, FoldMason itself relies on pairwise alignments during its initial guide-tree construction stage, so improvements in pairwise alignment sensitivity directly benefit the downstream MSA. To assess this concretely, we incorporated LoL-align into FoldMason's initial alignment stage and benchmarked the resulting MSAs on HomStrad and AFDB. LoL-align-initialized FoldMason achieves a higher overall F1 (69.62 vs. 69.50), with the gain concentrated on HomStrad (F1 78.31 vs. 77.98) where remote-homology cases are most prevalent. This demonstrates that the two methods are synergistic: LoL-align can improve the pairwise alignments on which FoldMason builds.

Based on these results, we recommend the following workflow:

- For **pairwise comparisons** or settings where only a few related structures are available (e.g., domain-boundary refinement between two extremely remote homologs), LoL-align is the more appropriate choice.
- For **multiple structure alignment** of a set of putatively related structures, FoldMason should be used.
- The two approaches can also be combined: LoL-align can be used to identify candidate remote homologs (e.g., for inclusion in an MSA), and FoldMason can then refine the joint alignment using the broader evolutionary context.

We have added this guidance to the Discussion section to clarify the intended usage workflow as follows:

“The integration of LoL-align into Foldseek facilitates its integration with other tools within the Foldseek ecosystem, such as Foldmason, for which replacing TMalign by LoL-align resulted in slight improvements (Table S1)”

	homstrad			afdb			overall
	prec	sens	f1	lddt	osc	f1	f1
+LoLalign	78.2145	78.4109	78.3126	60.3907	65.1144	62.6637	69.6196
-LoLalign	77.9218	78.0327	77.9772	60.4139	65.145	62.6903	69.5031

4. On page 6, the LoL-score is trained on the alignment results of TM-align. Since the training labels originate from TM-align, is LoL-align essentially "learning and optimizing" the behavior of TM-align? The authors are encouraged to discuss how LoL-align achieves results that exceed (rather than merely approximate) its training baseline.

We thank the reviewer for raising this important point, which allows us to clarify a critical aspect of our methodology and correct an inaccuracy in our original manuscript.

First, we clarify that the LoL-score network is trained on alignments generated by **Foldseek**, rather than TM-align. We have corrected this phrasing on page 6 of the revised text.

However, the reviewer's core conceptual question—whether a model exceeds the baseline used to generate its training labels—remains relevant, and our architecture achieves this through two main mechanisms:

Decoupled Local Prediction and Global Assembly: The LoL-score network is not trained to mimic full, end-to-end Foldseek alignments. Instead, it functions strictly as a **per-residue local quality estimator**, predicting whether an isolated candidate residue pair is structurally reliable. The downstream alignment itself is constructed using LoL-align's own independent, global **seed-and-refine dynamic programming procedure**. Because the network provides a continuous landscape of local structural confidence rather than a rigid end-to-end path, our alignment engine can discover optimal, globally consistent correspondences that Foldseek misses. This is especially pronounced in the remote-homology regime, where Foldseek's discrete 3Di structural alphabet encounters sensitivity limits that our continuous scoring bypasses.

Network Capacity as a Geometric Regularizer: The LoL-score network is intentionally designed with a low-capacity architecture. By deliberately constraining the model's size, we prevent it from memorizing the specific alignment errors, artifacts, or noise present in individual Foldseek outputs. Instead, the network is mathematically forced to capture only the consistent, generalizable geometric signals shared across the vast training set. In this framework, occasional baseline misalignments act as zero-mean noise that is effectively filtered out during optimization.

We corrected our mistake and replaced TM-align by Foldseek and included a discussion of these points in the Manuscript:
"Since potential Foldseek misalignments constitute only a small fraction of the

training data, the small network learns the dominant signal distinguishing homologous from non-homologous residue pairs rather than fitting to these errors.”

5. On Page 8, although the authors emphasize that the choice of 3Di weight (2.1) and AA weight (1.4) follows the methodological settings of Foldseek, is there a risk of overfitting these parameters to specific datasets used in that study, such as SCOPe? Evidence of generalizability to more diverse or recently predicted structural databases would be beneficial.

We thank the reviewer for pointing out that parameters inherited from prior work should be tested for robustness on the dataset at hand.

To assess the sensitivity of the 3Di-to-AA weight ratio, we evaluated three ratios — 1.4:1.4, 2.1:1.4 (current), and 2.8:1.4 — on the SCOPe all-vs-all benchmark using the Foldseek prefilter. The differences across all three settings are small (≤0.7 percentage points at the family, superfamily, and fold levels), indicating that LoL-align is not finely tuned to a specific ratio and that the chosen setting does not yield an overfit advantage on SCOPe.

Generalizability beyond SCOPe is further demonstrated by our full-length AlphaFold benchmark. LoL-align maintains its performance advantage in this independent setting, supporting that the parameter choices generalize beyond the SCOPe dataset.

We included the following table in the Supplementary and mentioned it in the text as follows:

“To verify that this weighting does not overfit to a particular dataset, we evaluated alternative ratios on SCOPe (Table S2).”

	**Family**	**Super Family**	**Fold**
1.4:1.4	0.885364	0.660518	0.214206
1.4:2.1	0.891	0.66584	0.2112
2.8:1.4	0.884126	0.659299	0.213696

Reviewer #1

This manuscript reports a highly sensitive and fast probabilistic tool (LoL-align) for protein structure alignment. The tool has the following major advantages or innovations:

First, it is not only more sensitive in identifying homologous proteins than other widely used tools for protein structure alignment, including DALI and TM-align, but also much faster than them (20 times faster than DALI and five times faster than TM-align). Moreover, the quality of alignments generated by LoL-align is substantially better than that of the other tools. Therefore, LoL-align is very useful for protein structure alignment and search, which has become indispensable for biological research as a vast amount of high-quality predicted protein structures are available nowadays.

Second, a Local-Log-odds scoring function used in LoL-align is novel. The novel score is a key reason that it achieves better sensitivity than other methods.

Third, a new vectorized Forward–Backward probabilistic sequence alignment method that can leverage GPU computing architecture is developed, which makes it much faster than DALI and TM-align.

Fourth, the tool has been integrated with a widely used, super-fast protein structure database search tool – FoldSeek, making it much easier for users to use it. This can greatly increase the impact of the tool.

In addition to the significant advantages above, the experiment in this work is well designed. The results are rigorously evaluated.

Overall, LoL-align represents a very significant, much-needed advance in the field of protein structure alignment and search and is expected to have a strong impact on protein structure prediction, analysis, and usage.

Below are a few minor comments for the authors to consider:
(1) In this work, SCOPe40 (11,211 proteins clustered at 40% sequence identity from SCOPe 2.01) was used for evaluation. The query set comprised 3,566 structures, restricted to SCOPe entries with at least one additional family-, superfamily-, or fold-level member. What is the distribution of the sequence identity between the proteins in the evaluation set (11,211 proteins) and those in the query set?

How does LoL-align's performance change when the sequence identity between the proteins in the query set and the ones in the evaluation set changes from high to low? The answers to these questions can help users understand how well LoL-align works when the sequence similarity between query proteins and target proteins is low.

We thank the reviewer for this insightful suggestion. To address it, we (i) computed the distribution of pairwise sequence identities between each query and its true-positive targets at the family, superfamily, and fold levels, and (ii) stratified the per-query ROC1 sensitivity by

the mean sequence identity of each query's same-superfamily cross-family targets. Sequence identities are derived from the structural alignments themselves; we report both LoL-align-based and TM-align-based binnings to ensure the analysis is not biased toward any single method.

The distributions confirm that SCOPe40 is dominated by remote homologs: at the superfamily level, the distribution peaks around 10–15% identity and decays rapidly above 25%, with essentially no pairs exceeding 40%. The fold-level distribution is shifted even further toward low identity, peaking around 8–12%. As expected, family-level pairs are slightly more similar but still concentrated in the twilight zone (peak ~15–20%). Aggregated into bins, the LoL-align-based binning yields n = 882, 2,284, 329, and 71 queries for the 0–10%, 10–20%, 20–30%, and ≥30% bins, respectively; the TM-align-based binning gives n = 2,344, 1,029, 145, and 46.

We added three plots to the Supplementary (S6-S8) and referred to them in the text as: “We also analyzed the performance on different sequence identity levels, see Figures. S6-S8”

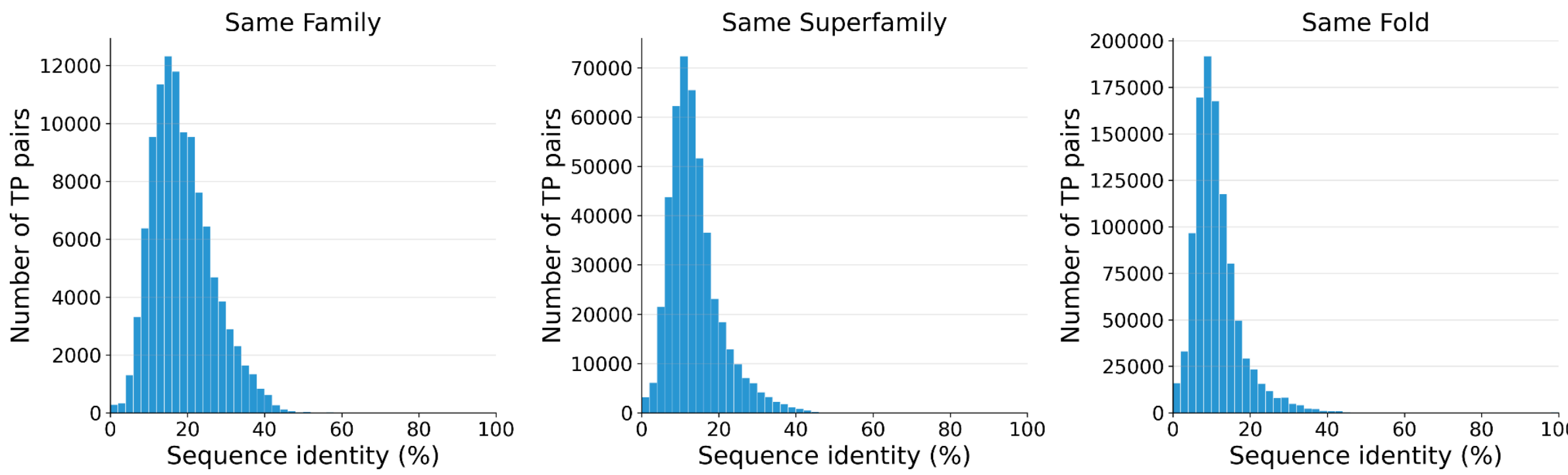

LoL-align Binning:

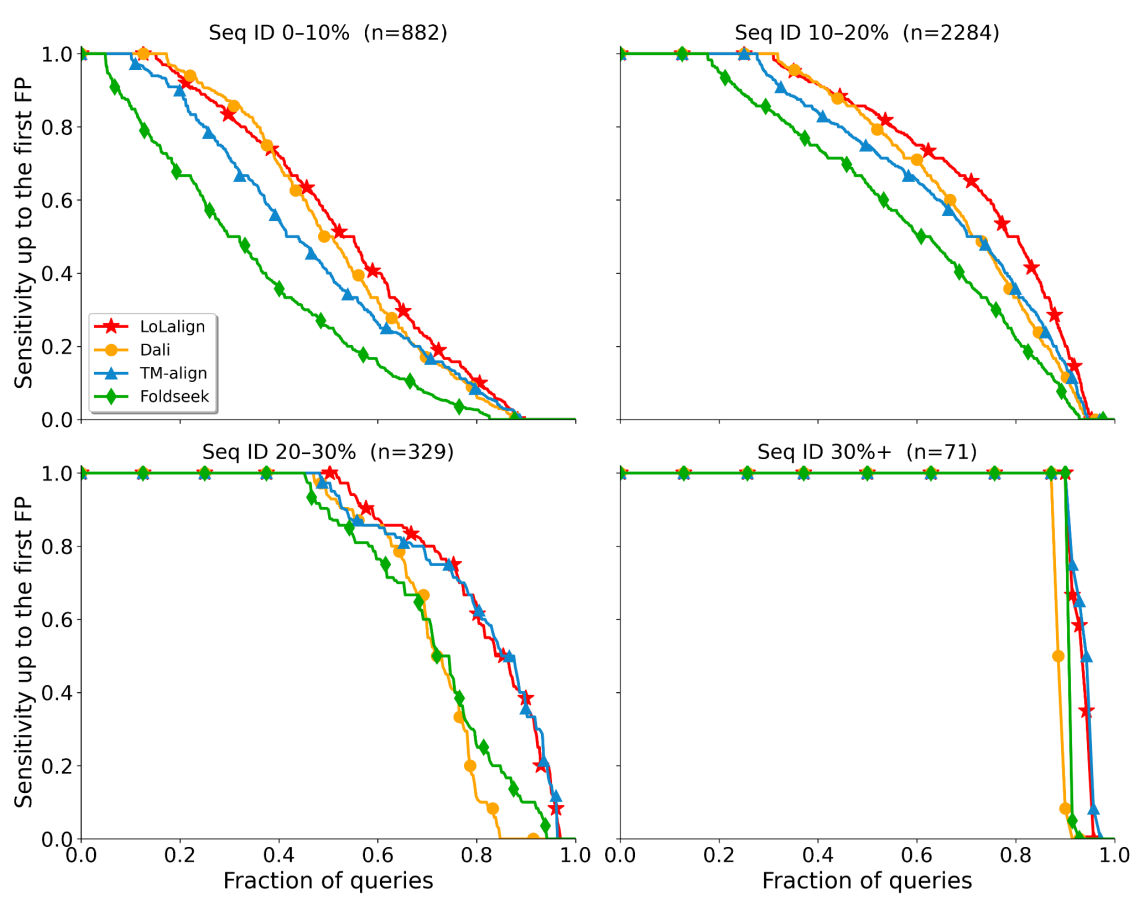

TM-align Binning:

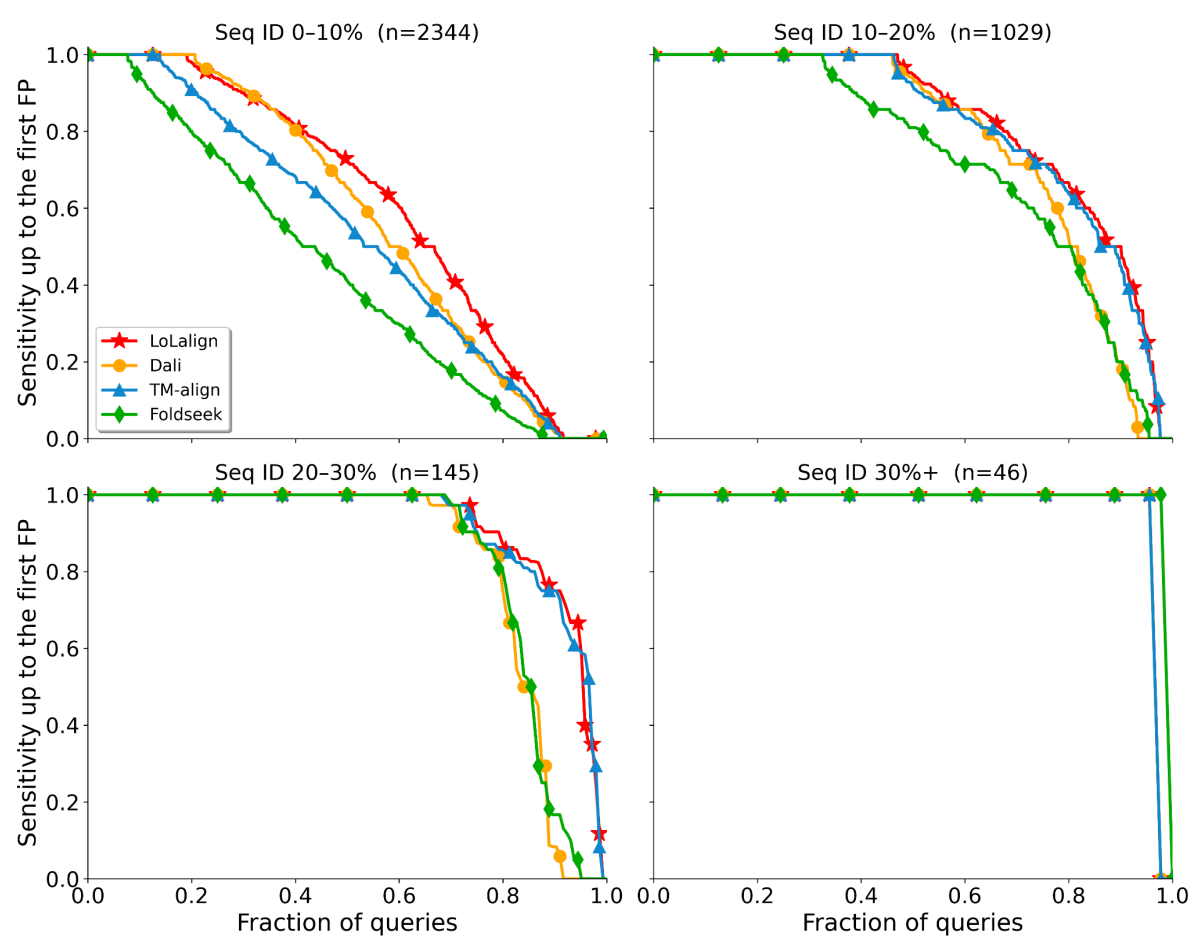

(2)Please discuss if it is possible to assign a statistical significance score (e.g., p-value) to the alignment score calculated by LoL-align.

We thank the reviewer for this insightful suggestion. Providing statistical significance estimates (such as e-values or p-values) is indeed a highly valuable feature for ranking and interpreting hits, particularly in large-scale structural database searches.

In principle, an e-value can be assigned to LoL-align scores by fitting an Extreme Value Distribution (EVD) to a background distribution of unrelated structure pairs—factoring in query and target lengths—as is standard practice in sequence and structure search tools like BLAST and Foldseek. While this is straightforward on curated datasets like SCOPe (where cross-fold pairs provide a clean, ground-truth negative set), doing so for full-length proteins in databases like the AlphaFold Database (AFDB) presents a major bottleneck. Without manual annotations, partitioning full-length structures into "related" and "unrelated" creates a circular dependency on the structural aligner itself, and an EVD fitted on single-domain SCOPe structures does not generalize well to multi-domain, full-length data.

To address this, we extensively explored methods to generate a reliable full-length null model. In sequence alignment, a common strategy for E-value calibration is database reversal. To translate this concept to structure space, we attempted to generate physical decoy structures by reversing C-alpha trajectories and reconstructing the backbones using a variety of methods—ranging from classical tools like PULCHRA to advanced deep-learning approaches like AlphaFold's initial guess and Cg2all. We then evaluated whether these reconstructed decoys could replicate the empirical false-positive (cross-fold) distribution on SCOPe. Unfortunately, the reversed structures consistently failed to model the true background distribution, performing significantly worse than the original cross-fold negatives. To our knowledge, a robust method for generating a realistic null model for full-length structural data remains an open challenge in the field.

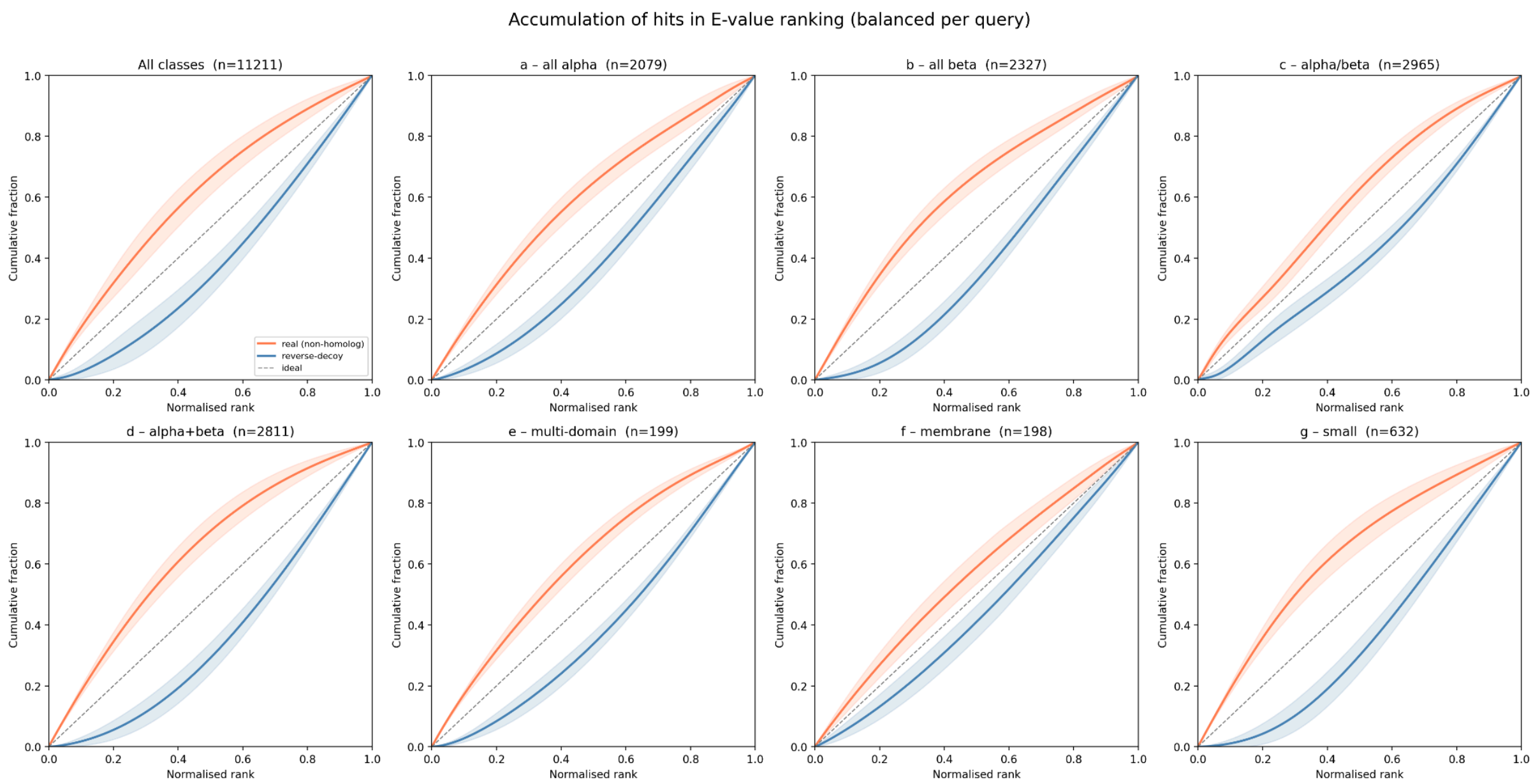

However, we entirely agree with the reviewer that an interpretable metric is necessary to help users distinguish true homologs from non-homologs. To solve this, we have introduced a lightweight neural network that predicts a homology probability ("LoL-align probability") based solely on local alignment features. We trained this model on SCOPe, defining superfamily members as true positives and cross-fold pairs as false positives. By strictly restricting the inputs to local structural features, the network can bypass global length artifacts and generalize well to full-length proteins.

We have rerun our full-length benchmark using this new probability score and observed an interesting trade-off: while the standard LoL-align score prioritizes global structural similarity (yielding higher sensitivity), the LoL-align probability score prioritizes shorter, high-quality local alignments, boosting precision on the benchmark. We believe this addition directly addresses the reviewer's core concern by providing users with an easily interpretable "probability of homology".

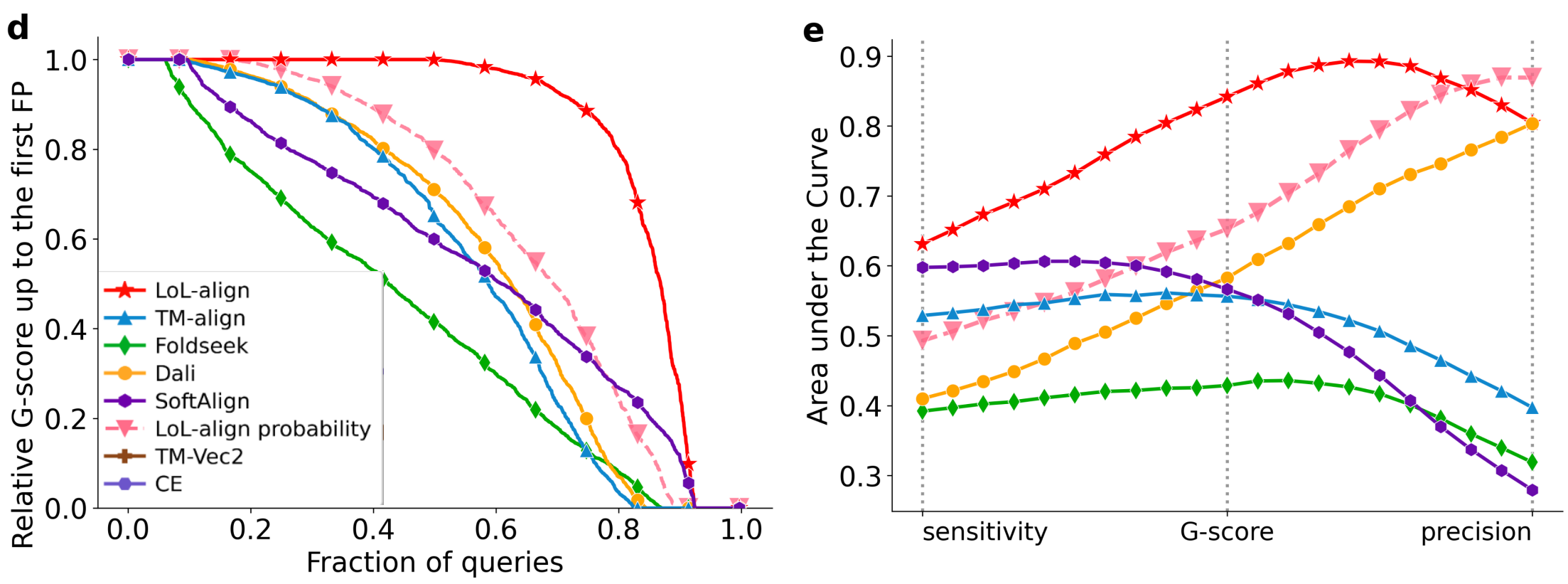

We added a new section in the Online method part, where we describe features, training, etc., and added a Cross-Validation benchmark in the Supplementary (S9).

We also mention the new Score in the main text as follows:

While the LoL-score reflects global similarity of two aligned proteins, it is less suited for determining whether a local alignment represents true homology. We therefore trained a small neural network on the SCOPe database to predict the probability of a homologous match from only local alignment features. On the single-domain benchmark, the probability performs similarly to the LoL-score (Fig. S9), while on the full-length benchmark, it places more emphasis on residue-wise precision than sensitivity (Fig 2d,e), Fig. S4.

(3) In this sentence, "we selected 2,000 structures from the 2.3 million cluster representatives
in the clustered AFDB and sampled 2000 query proteins with at least 100 residues scoring ≥ 0.7 predicted Local Distance Difference Test (pLDDT) score", are the 2000 structures and the sampled 2000 query proteins the same thing? Right now, they are described separately, making it read like the 2000 query proteins are sampled from the 2000 structures. If they refer to the same 2000 proteins, the sentence can be revised to reflect it. For instance, it can

be revised as "We identified 2,000 structures from the AFDB's 2.3 million cluster representatives as query proteins, requiring each structure to have a minimum of 100 residues with a pLDDT score ≥ 0.7.".

We thank the reviewer for pointing out this ambiguity. The reviewer is correct that the 2,000 structures and the 2,000 query proteins refer to the same set. We have revised the sentence in the manuscript to make this explicit.

Reviewer #2

This study introduces LoL-align, a novel protein structure alignment algorithm that leverages a Local Log-odds scoring function and a vectorized Forward-Backward algorithm to achieve a balance between sensitivity and computational speed. The benchmarking results on SCOPe and AFDB datasets demonstrate that LoL-align offers superior sensitivity for remote homologs compared to traditional tools like TM-align and Dali while being significantly faster. However, while the work is solid, several critical aspects regarding the algorithm's robustness, the scope of benchmarking, and its relationship with existing methodologies require further clarification and refinement.
1. The algorithm may be highly dependent on the initial "seed anchors." If the initial 7-mer seed is chosen incorrectly, will the iterative process fall into a local optimum or lead to erroneous alignments? I suggest that the authors supplement a sensitivity analysis on the impact of seed selection on the final alignment quality, particularly for proteins with numerous low-pLDDT regions.

We thank the reviewer for raising this important point. To address it, we have made the seed-selection step user-configurable by exposing two parameters: `--candidate-seeds`, the number of candidate seeds for which an ungapped structural alignment is computed, and `--refine-seeds`, the number of top-ranked seeds that are subsequently refined. This allows the trade-off between robustness against suboptimal seeds and runtime to be tuned explicitly.

To quantify the sensitivity to seed selection, we evaluated `--refine-seeds` values of 1, 3, 5, and 10 on both the SCOPe all-vs-all benchmark and the full-length AlphaFold benchmark, which includes proteins with extensive low-pLDDT regions. On SCOPe, performance increases monotonically with the number of refined seeds and begins to saturate around 5–10 seeds. On the full-length benchmark, increasing the number of refined seeds yields consistent improvements across all evaluated

metrics — G-score, LDDT normalized by alignment length (precision), LDDT normalized by query length (sensitivity), and overall alignment quality — for proteins with both high- and low-pLDDT regions.

We describe these features in the Online Method section as follows

“Because the final alignment depends on the selected anchors, we expose two user-configurable parameters: the number of candidate seeds for which an ungapped structure alignment is computed --candidate-seeds and the number subsequently refined top-ranked seeds --refine-seeds. The effect of --refine-seeds is reported in Table S2 and Figures S4 and S5.”

The New Low plddt benchmark is mentioned as follows:

“To evaluate performance on low-confidence regions, we additionally sampled 1000 queries containing at least 100 residues with pLDDT less than or equal to 0.5. Results for the high- and low-confidence sets are reported in Figures S4 and S5, respectively.”

In addition, to further reduce the risk of selecting spurious seeds in unstructured regions, we had re-fitted the 3Di substitution matrix on TM-align alignments from the AlphaFold database. The re-fitted matrix assigns lower scores to matches in unstructured regions than the original SCOPe-trained matrix, making low-pLDDT segments less likely to be chosen as initial anchors.

The new matrix is described in the Online Method part like this, and the Substitution matrix is shown in the supplementary:

“Furthermore, since the original 3Di substitution matrix was fitted on SCOPe and assigns high scores to matches in unstructured regions, we instead use a 3Di matrix re-fitted on TM-align alignments from the AlphaFold database, which yields lower scores for such spurious matches (Table S4)”

High pLDDT:

Each column has a different true positive cutoff. Left 0.4, middle 0.5, right 0.6.

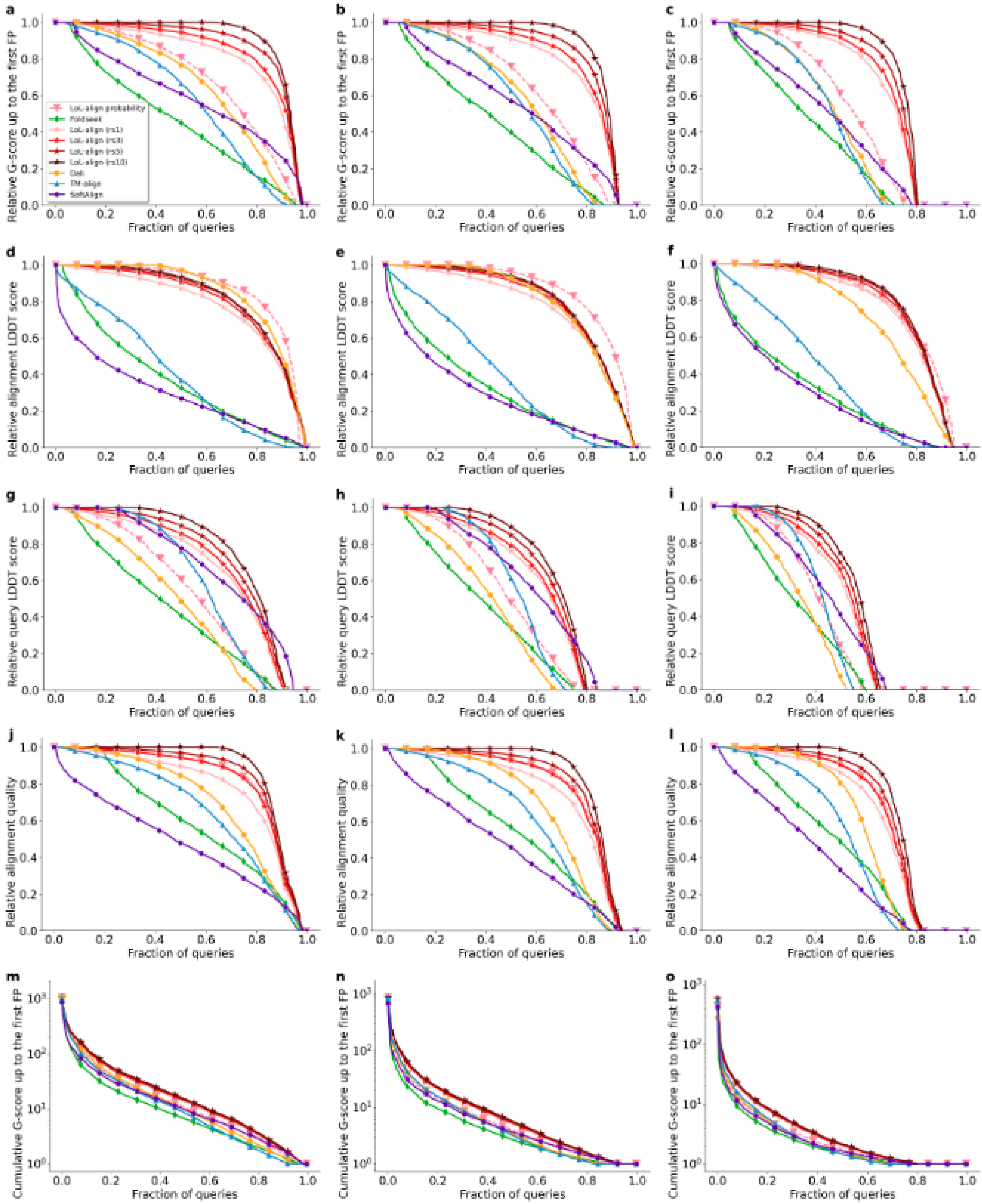

Low pLDDT:

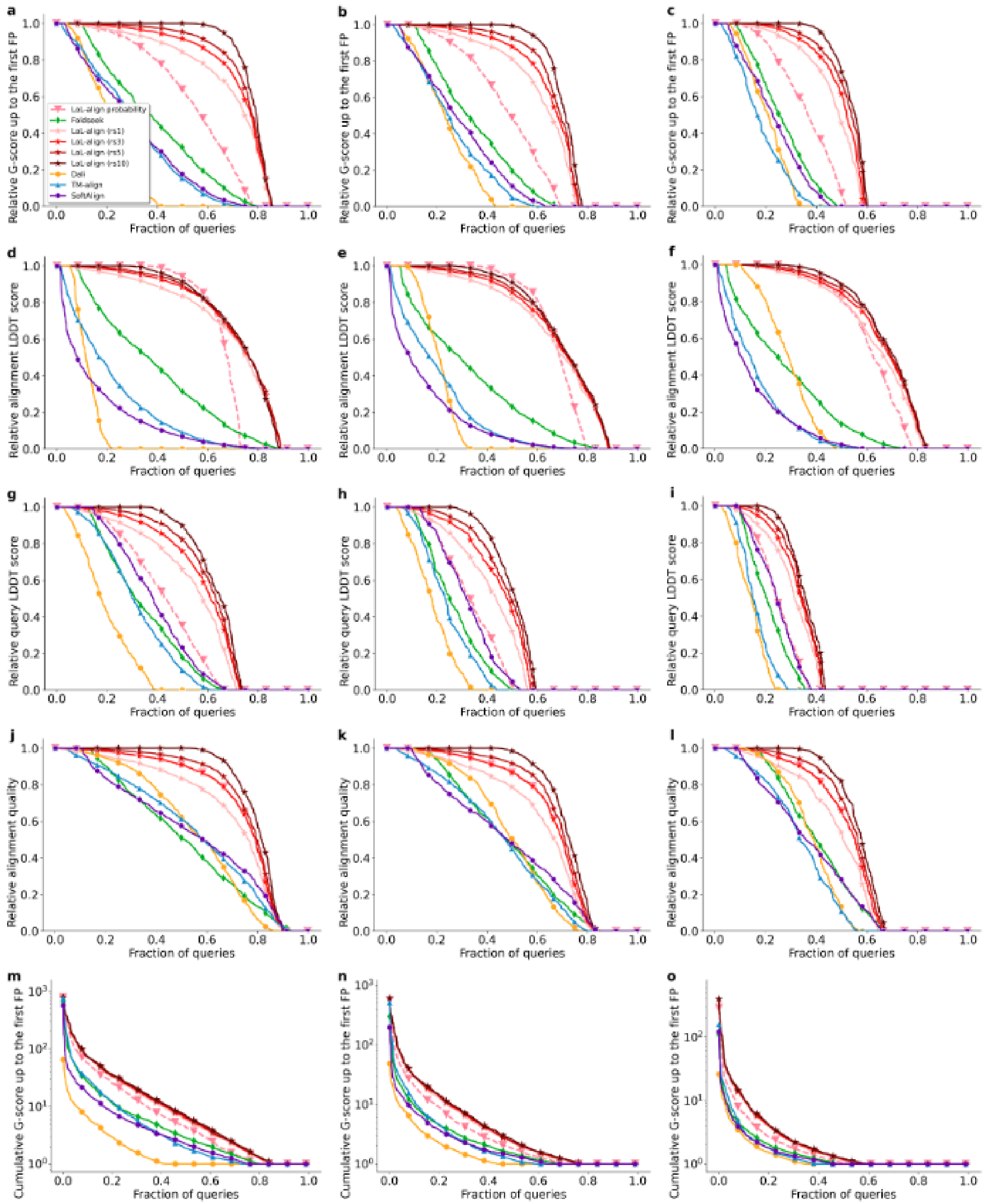

a
b
c
d
e
f
g
h
i
j
k
l
m
n
o
Relative G-score up to the first FP
Relative alignment LDDT score
Relative query LDDT score
Relative alignment quality
Cumulative G-score up to the first FP
Fraction of queries
LoL-align probability
Foldseek
LoL-align (rs1)
LoL-align (rs3)
LoL-align (rs5)
LoL-align (rs10)
Dali
TM-align
SoftAlign

2. The authors mention that deep learning methods struggle when processing proteins outside their training distribution; however, the benchmarks primarily compare LoL-align with traditional tools such as TM-align and Dali. In recent years, many structural alignment or search tools based on deep learning have emerged, such as TM-Vec and DeepBLAST. Comparing only with traditional algorithms may not fully demonstrate the competitiveness of LoL-align. It is recommended to supplement the performance comparison with representative deep learning alignment tools.

We thank the reviewer for this suggestion. We have added two recent deep-learning-based methods to our benchmarks: **SoftAlign**, a neural structural aligner, and **TM-Vec2**, a sequence-based structural search method.

SoftAlign has been added to the full-length AlphaFold benchmarks alongside the existing baselines. It is worth mentioning that Soft-align uses a soft LDDT score to rank the alignments by default, which can boost the performance on this benchmark

TM-Vec2 operates on sequence input only and produces per-structure embeddings for retrieval rather than residue-level alignments. We therefore included it only in the SCOPe all-vs-all benchmark, where retrieval performance can be compared directly. LoL-align achieves higher sensitivity than TM-Vec2, particularly at the superfamily and fold levels, consistent with the expectation that structure-based aligners retain an advantage over sequence-only methods in the remote-homology regime that dominates SCOPe40.

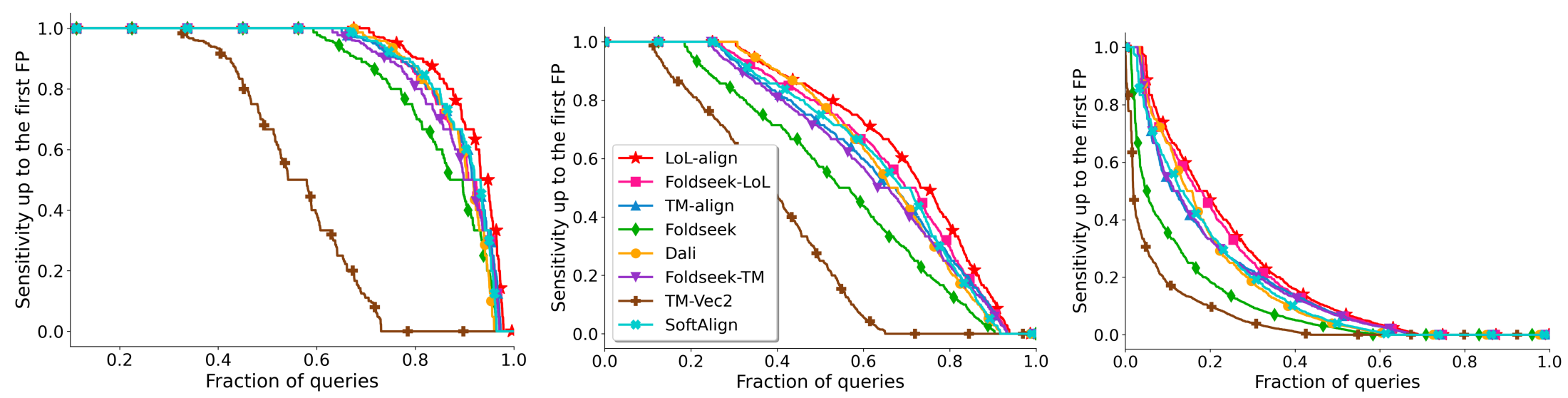

3. LoL-align emphasizes its sensitivity advantage in detecting remote homology. Given that FoldMason can capture more conserved evolutionary signals through multi-sequence information, which approach is more accurate for defining domain boundaries when dealing with extremely remote homology pairs: pairwise refinement using LoL-align or direct MSA construction using FoldMason? Do the authors have clear recommendations for the usage workflow? I believe providing these clarifications is crucial for further promoting the broad adoption of the tool.

We thank the reviewer for this insightful question, which touches on an important methodological distinction.

LoL-align and FoldMason address related but distinct problems. LoL-align is a *pairwise* structural aligner optimized for sensitivity in the remote-homology regime, while FoldMason is a *multiple* structure aligner that leverages information across many homologs simultaneously, which can indeed provide a stronger evolutionary signal when sufficiently many related structures are available. The two approaches are therefore complementary rather than alternative options.

Importantly, FoldMason itself relies on pairwise alignments during its initial guide-tree construction stage, so improvements in pairwise alignment sensitivity directly benefit the downstream MSA. To assess this concretely, we incorporated LoL-align into FoldMason's initial alignment stage and benchmarked the resulting MSAs on HomStrad and AFDB. LoL-align-initialized FoldMason achieves a higher overall F1 (69.62 vs. 69.50), with the gain concentrated on HomStrad (F1 78.31 vs. 77.98) where remote-homology cases are most prevalent. This demonstrates that the two methods are synergistic: LoL-align can improve the pairwise alignments on which FoldMason builds.

Based on these results, we recommend the following workflow:

- For **pairwise comparisons** or settings where only a few related structures are available (e.g., domain-boundary refinement between two extremely remote homologs), LoL-align is the more appropriate choice.
- For **multiple structure alignment** of a set of putatively related structures, FoldMason should be used.
- The two approaches can also be combined: LoL-align can be used to identify candidate remote homologs (e.g., for inclusion in an MSA), and FoldMason can then refine the joint alignment using the broader evolutionary context.

We have added this guidance to the Discussion section to clarify the intended usage workflow as follows:

“The integration of LoL-align into Foldseek facilitates its integration with other tools within the Foldseek ecosystem, such as Foldmason, for which replacing TMalign by LoL-align resulted in slight improvements (Table S1)”

	homstrad			afdb			overall
	prec	sens	f1	lddt	osc	f1	f1
+LoLalign	78.2145	78.4109	78.3126	60.3907	65.1144	62.6637	69.6196
-LoLalign	77.9218	78.0327	77.9772	60.4139	65.145	62.6903	69.5031

4. On page 6, the LoL-score is trained on the alignment results of TM-align. Since the training labels originate from TM-align, is LoL-align essentially "learning and optimizing" the behavior of TM-align? The authors are encouraged to discuss how LoL-align achieves results that exceed (rather than merely approximate) its training baseline.

We thank the reviewer for raising this important point, which allows us to clarify a critical aspect of our methodology and correct an inaccuracy in our original manuscript.

First, we clarify that the LoL-score network is trained on alignments generated by **Foldseek**, rather than TM-align. We have corrected this phrasing on page 6 of the revised text.

However, the reviewer's core conceptual question—whether a model exceeds the baseline used to generate its training labels—remains relevant, and our architecture achieves this through two main mechanisms:

Decoupled Local Prediction and Global Assembly: The LoL-score network is not trained to mimic full, end-to-end Foldseek alignments. Instead, it functions strictly as a **per-residue local quality estimator**, predicting whether an isolated candidate residue pair is structurally reliable. The downstream alignment itself is constructed using LoL-align's own independent, global **seed-and-refine dynamic programming procedure**. Because the network provides a continuous landscape of local structural confidence rather than a rigid end-to-end path, our alignment engine can discover optimal, globally consistent correspondences that Foldseek misses. This is especially pronounced in the remote-homology regime, where Foldseek's discrete 3Di structural alphabet encounters sensitivity limits that our continuous scoring bypasses.

Network Capacity as a Geometric Regularizer: The LoL-score network is intentionally designed with a low-capacity architecture. By deliberately constraining the model's size, we prevent it from memorizing the specific alignment errors, artifacts, or noise present in individual Foldseek outputs. Instead, the network is mathematically forced to capture only the consistent, generalizable geometric signals shared across the vast training set. In this framework, occasional baseline misalignments act as zero-mean noise that is effectively filtered out during optimization.

We corrected our mistake and replaced TM-align by Foldseek and included a discussion of these points in the Manuscript:
"Since potential Foldseek misalignments constitute only a small fraction of the

training data, the small network learns the dominant signal distinguishing homologous from non-homologous residue pairs rather than fitting to these errors.”

5. On Page 8, although the authors emphasize that the choice of 3Di weight (2.1) and AA weight (1.4) follows the methodological settings of Foldseek, is there a risk of overfitting these parameters to specific datasets used in that study, such as SCOPe? Evidence of generalizability to more diverse or recently predicted structural databases would be beneficial.

We thank the reviewer for pointing out that parameters inherited from our prior work should be tested for robustness on the dataset at hand.

To assess the sensitivity of the 3Di-to-AA weight ratio, we evaluated three ratios — 1.4:1.4, 2.1:1.4 (current), and 2.8:1.4 — on the SCOPe all-vs-all benchmark using the Foldseek prefilter. The differences across all three settings are small (≤0.7 percentage points at the family, superfamily, and fold levels), indicating that LoL-align is not finely tuned to a specific ratio and that the chosen setting does not yield an overfit advantage on SCOPe.

Generalizability beyond SCOPe is further demonstrated by our full-length AlphaFold benchmark. LoL-align maintains its performance advantage in this independent setting, supporting that the parameter choices generalize beyond the SCOPe dataset.

We included the following table in the Supplementary and mentioned it in the text as follows:

“To verify that this weighting does not overfit to a particular dataset, we evaluated alternative ratios on SCOPe (Table S2).”

	**Family**	**Super Family**	**Fold**
1.4:1.4	0.885364	0.660518	0.214206
1.4:2.1	0.891	0.66584	0.2112
2.8:1.4	0.884126	0.659299	0.213696

Second Round
Reviewer #1

The revised manuscript addressed all my previous comments satisfactorily. The method is a significant contribution to protein structure alignment and can have broad application in the field.

Reviewer #2

I thank the authors for their careful and constructive revision. In the revised manuscript and response letter, the authors have adequately addressed my previous concerns. In particular, the additional clarifications, analyses, and revisions have improved the rigor and transparency of the study. I am satisfied with the authors' responses and do not have further major concerns.

Remarks on code availability:
I have reviewed the code-related materials.

We thank both reviewers for their time and constructive engagement with our manuscript throughout the review process.
